# A high-throughput approach to identify BRCA1-downregulating compounds to enhance PARP inhibitor sensitivity

**DOI:** 10.1016/j.isci.2024.110180

**Published:** 2024-06-04

**Authors:** Erin Sellars, Margarita Savguira, Jie Wu, Sabrina Cancelliere, Mark Jen, Rehna Krishnan, Anne Hakem, Dalia Barsyte-Lovejoy, Razqallah Hakem, Steven A. Narod, Joanne Kotsopoulos, Leonardo Salmena

**Affiliations:** 1 Department of Pharmacology & Toxicology, University of Toronto, Toronto, ON M5S 1A8, Canada; 2 Women’s College Research Institute, Women’s College Hospital, Toronto, ON M5S 1B2, Canada; 3 Lunenfeld-Tanenbaum Research Institute, Network Biology Collaborative Centre, High-Throughput Screening, Mt. Sinai Hospital, Sinai Health System, Toronto, ON M5G 1X5, Canada; 4 Princess Margaret Cancer Centre, University Health Network, Toronto, ON M5G 1L7, Canada; 5 Structural Genomics Consortium, University of Toronto, Toronto, ON M5G 1L7, Canada; 6 Department of Medical Biophysics, University of Toronto, Toronto, ON M5G 1L7, Canada; 7 Dalla Lana School of Public Health, University of Toronto, Toronto, ON M5T 3M7, Canada

**Keywords:** Pharmacology, Cell biology, Cancer

## Abstract

PARP inhibitors (PARPi) are efficacious in *BRCA1*-null tumors; however, their utility is limited in tumors with functional BRCA1. We hypothesized that pharmacologically reducing BRCA1 protein levels could enhance PARPi effectiveness in *BRCA1* wild-type tumors. To identify BRCA1 downregulating agents, we generated reporter cell lines using CRISPR-mediated editing to tag endogenous BRCA1 protein with HiBiT. These reporter lines enable the sensitive measurement of BRCA1 protein levels by luminescence. Validated reporter cells were used in a pilot screen of epigenetic-modifying probes and a larger screen of more than 6,000 compounds. We identified 7 compounds that could downregulate BRCA1-HiBiT expression and synergize with olaparib. Three compounds, N-acetyl-N-acetoxy chlorobenzenesulfonamide (NANAC), A-443654, and CHIR-124, were validated to reduce BRCA1 protein levels and sensitize breast cancer cells to the toxic effects of olaparib. These results suggest that BRCA1-HiBiT reporter cells hold promise in developing agents to improve the clinical utility of PARPi.

## Highlights

• PARP inhibitors (PARPi) are highly effective in cancers with a loss of BRCA1 protein
• Reducing BRCA1 expression with drugs could enhance the effectiveness of PARPi
• BRCA1-HiBiT reporter cells enabled the screening of over 6,000 compounds
• Three drugs were identified to decrease BRCA1 expression and synergyze with PARPi

## Graphical abstract

**Figure:**
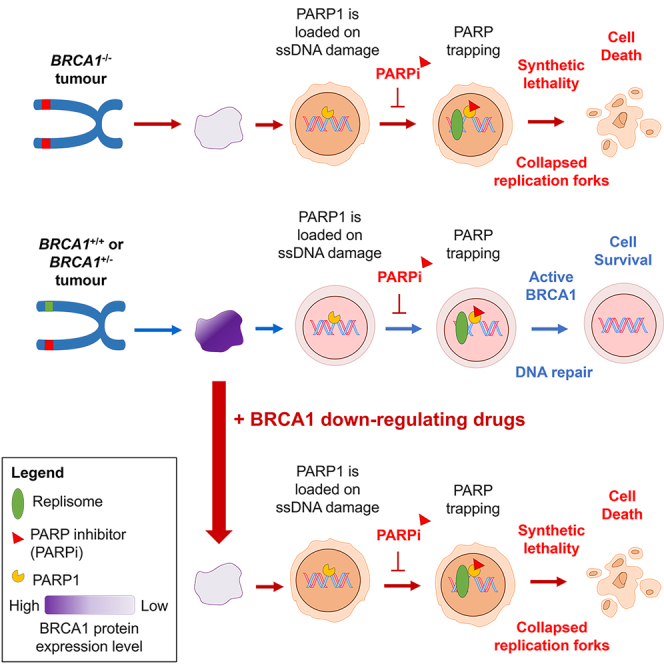

## Introduction

Mutations in the breast cancer susceptibility gene 1 (*BRCA1*) are associated with an increased risk of triple-negative breast cancer and ovarian cancer, both of which pose significant challenges in treatment due to their aggressive nature.^1^ The major cellular function of *BRCA1* is the repair of double-strand breaks (DSBs) through homologous recombination (HR).^2^ Cells that harbor loss-of-function *BRCA1* mutations exhibit increased genomic instability due to their inability to perform error-free HR, rendering them more susceptible to malignant transformation.^3^^,^^4^ Synthetic lethality describes a phenomenon in which a combination of two molecular perturbations is lethal, whereas each individual perturbation is well tolerated.^5^^,^^6^ *BRCA1*-associated synthetic lethality was first reported in 2005 when it was observed that *BRCA1*-deficient cells were exquisitely sensitive to the effects of inhibitors of poly(ADP-ribose)-polymerase (PARP).^7^^,^^8^ The PARP family of enzymes is essential for single-strand break repair and base excision repair (BER).^5^^,^^9^ PARP1 is an abundant nuclear protein and the most prominent member of the PARP family that is responsible for more than 80% of all cellular PARP activities.^5^^,^^10^ PARP1 recruitment is one of the earliest events in the DNA damage response (DDR).^5^^,^^6^^,^^10^ At the site of DNA damage, PARP1 is activated and PARylates itself, histones and other chromatin-associated proteins, which serve as a scaffold for the recruitment and assembly of other DNA repair proteins, thereby promoting effective repair of DNA.^10^^,^^11^^,^^12^ PARP inhibitors (PARPi) function by trapping the PARP1 enzyme on DNA, leading to the accumulation of unrepaired ssDNA breaks and stalled replication forks.^5^ In most normal cells expressing BRCA1, stalled replication forks generated by PARPi can be effectively resolved through the HR repair pathway. However, in HR-deficient or *BRCA1*-null cells, stalled replication forks lead to chromosomal instability, cell-cycle arrest, and eventual cell death.

PARPi have demonstrated therapeutic efficacy in cancers characterized as HR deficient, including those with mutations in *BRCA1*, *BRCA2*, or other genes associated with "BRCAness," such as *ATM*, *ATR*, *PALB2*, and *FANC8*.^12^^,^^13^ In clinical practice, PARPi are used to treat recurrent *BRCA1* and *BRCA2-*mutated ovarian cancer,^14^^,^^15^^,^^16^^,^^17^ metastatic pancreatic cancer,^18^ and castration-resistant prostate cancer.^19^ Additionally, PARPi were recently approved for treating metastatic, HER2-negative breast cancer associated with germline BRCA1 and BRCA2 mutations, based on findings from the OlympiAD^20^^,^^21^ and EMBRACA^22^ clinical trials. However, the clinical utility of PARPi in breast cancer management is limited only to those patients with germline *BRCA1/2* mutation, which accounts for only a small percentage (approximately 5–10%) of all breast cancer cases.^23^^,^^24^^,^^25^^,^^26^ Furthermore, the generation of resistance through the reactivation of HR through mutations or epigenetic regulation of *BRCA1*, and other "BRCAness'' genes is a significant challenge in PARPi therapy that has been observed in diverse cancers.^6^^,^^27^ Based on this clinical need, we hypothesized that the selective downregulation of BRCA1 protein levels could sensitize (or resensitize) cells to the effects of PARPi and thus improve clinical effectiveness.

To facilitate the discovery of compounds that reduce BRCA1 protein levels, we developed a unique cell reporter system expressing endogenous BRCA1 protein with a C-terminal HiBiT-tag. In this study, we present evidence demonstrating that BRCA1-suppressing agents may sensitize BRCA1-competent cancer cells to PARPi.

## Results

### Generation of breast cancer susceptibility gene 1-HiBiT reporter cell model

**Figure 1:**
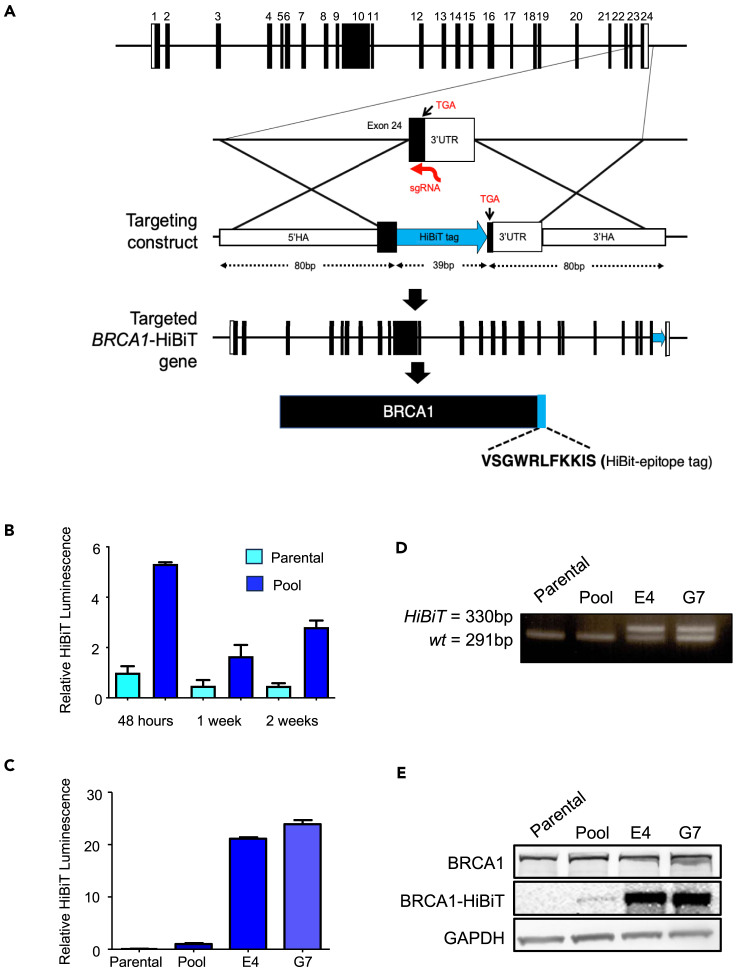
Targeting of *BRCA1* locus with HiBiT (A) A synthetic guide RNA (sgRNA) was identified that directed CRISPR-Cas9 to generate a DSB upstream of the BRCA1 stop codon within (the terminal) exon 24 of the BRCA1 gene. A targeting-donor construct was designed as a single-stranded oligonucleotide donor (ssODN) with 80bp 5′ and 3′ homology arms (HAs) that flank the repair sequence. Following the successful recombination of the targeting construct, the targeted BRCA1-HiBiT gene will express the full-length BRCA1-HiBiT fusion protein from the endogenous BRCA1 promoter locus. (B) Elevated luminescence persisted in targeted HEK293T cells over a period of 2 weeks and beyond. Data are represented as mean ± SEM. (C) HiBiT clones E4 and G7 have 25-fold greater luminescence signal than the heterogeneous pool. Relative HiBiT Luminescence represents luminescence normalized to parental cells. Data are represented as mean ± SEM. (D) Correct insertion of the HiBiT sequence was verified by PCR screening. HEK293T and HiBiT-targeted cells demonstrate 291bp WT amplicon whereas both E4 and G7 clones show a demonstrate WT and targeted amplicon (330bp). (E) Expression of the BRCA1-HiBiT protein was confirmed by Nano-Glo HiBiT Blotting. All replicated experiments were performed at least three times. See also Figures S1 and S2.

To effectively screen for drugs that reduce BRCA1 levels in a high-throughput manner, a robust cell model was necessary but unavailable. Although existing models such as transcriptional reporters, the ectopic expression of fluorescence-tagged proteins, and downstream sensors can be useful to explore changes in gene expression, protein stability, or function, they do not accurately capture all facets of gene regulation. To overcome this limitation, we generated reporter cell lines that express an endogenously tagged BRCA1 fusion protein, which preserves the normal regulatory environment of *BRCA1* at 17q21.31. To generate these reporter lines, we employed HiBiT, an 11-amino acid peptide that can interact with high affinity to LgBit to enable the conversion of furimazine to furimamide, resulting in bright luminescence.^28^^,^^29^ CRISPR-editing was utilized to facilitate the insertion of the *HiBiT* DNA sequence oligonucleotides at the extreme 3′-terminus of the *BRCA1* coding sequence, upstream of the stop codon in exon 24 in various cell lines, including the HEK293T, HeLa, DU145, U2OS, and MCF-7 cells (Figure 1A).

Among the transfected cell lines, HiBiT luminescence persisted beyond 2 weeks post-transfection, suggesting stable integration only in HEK293T and HeLa cells (Figure 1B; Figure S1A). Stable integration was not achieved, despite numerous attempts, in other cell lines (Figure S1A). Clonal selection of HiBiT expressing HEK293T cells yielded several clones including E4 and G7, which displayed more than a 20-fold increase in HiBiT luminescence compared to the targeted pool (Figure 1C). To confirm the correct integration of the *HiBiT* DNA sequence at the *BRCA1* stop codon, PCR using primers in the HiBiT flanking regions of the *BRCA1* locus was performed on genomic DNA isolated from clones E4, G7, the targeted pool, and parental HEK293T cells (Figure 1D; Figure S1B). Compared to the 291 bp PCR amplicon generated for the wild-type *BRCA1* allele, the *HiBiT* targeted *BRCA1* allele generated a larger amplicon of 330 bp (Figure 1D). The 291 bp *wild-type* product was amplified in all samples*,* whereas only the targeted pool and clones E4 and G7 generated an additional 330 bp amplicon representative of correct targeting. Sanger sequencing of the targeted allele confirmed correct recombination with no sequence errors at the 5′ and 3′ insertion junctions (Figures S1C and S1D). LgBiT-blotting demonstrated the presence of a 220 kDa HiBiT-tagged protein in the targeted pool and clones E4, G7 (Figure 1E) which corresponds with the full-length BRCA1 protein. These data confirm the generation of clonal populations of HEK293T cells with the heterozygous insertion of the HiBiT tag and correct expression of the full-length BRCA1-HiBiT fusion protein. We were also successful in generating two HiBiT-HeLa cell clones where F5 is heterozygous for the *BRCA1-HiBiT* allele and F11 is homozygous for the *BRCA1-HiBiT* allele (Figures S1A, S2A, and S2B).

### Breast cancer susceptibility gene -HiBiT reporter cells permit the sensitive measurement of breast cancer susceptibility gene 1 levels

**Figure 2:**
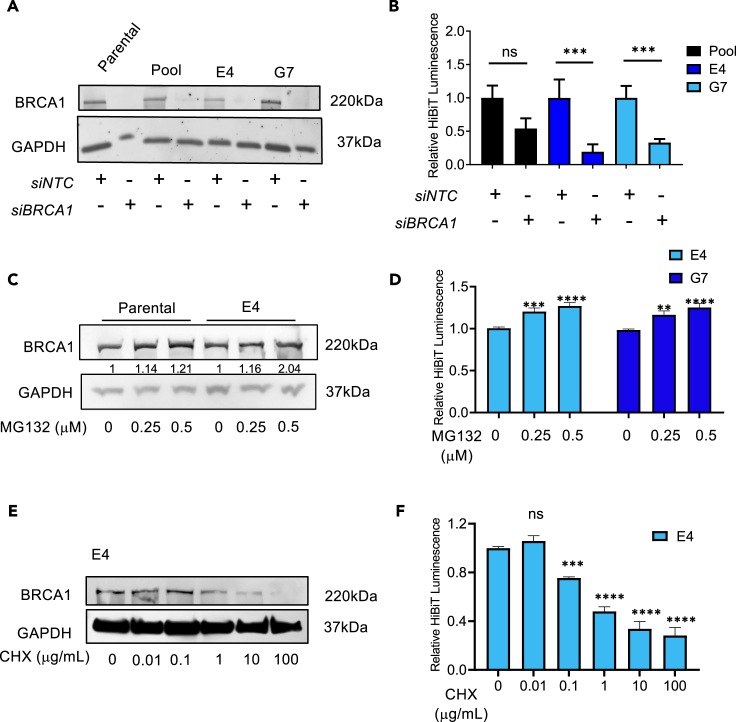
Validation of BRCA1-HiBiT reporter cells (A) BRCA1 Western blot demonstrating siRNA knockdown of *BRCA1*. (B) Histograms demonstrating HiBiT luminescence in HiBiT-targeted pool and both E4 and G7 clones transfected with BRCA1 siRNA or NTC siRNA. Relative HiBiT Luminescence refers to light units normalized to siNTC within the pool, E4, or G7 cell line. Data are represented as mean ± SEM. ∗ p<0.05, ∗∗ p<0.01, ∗∗∗ p<0.001, ∗∗∗∗ p<0.0001, ns, non-significant. (C) BRCA1 Western blot demonstrating HEK293T cells and BRCA1-HiBiT-clone E4 after treatment with the proteasome inhibitor, MG132. (D) Histograms demonstrating HiBiT luminescence E4 and G7 clones after 24h treatment of HEK293T cells with MG132. Relative HiBiT Luminescence refers to light units normalized to DMSO treated control within the E4, or G7 cell line. Data are represented as mean ± SEM. ∗ p<0.05, ∗∗ p<0.01, ∗∗∗ p<0.001, ∗∗∗∗ p<0.0001, ns, non-significant. (E) BRCA1 Western blot demonstrating BRCA1-HiBiT-clone E4 after treatment with cycloheximide. (F) Histograms demonstrating HiBiT luminescence in E4 clone after treatment with depicted doses of cycloheximide. Relative HiBiT Luminescence refers to light units normalized to DMSO treated control within the E4 cell line. Data are represented as mean ± SEM. ∗ p<0.05, ∗∗ p<0.01, ∗∗∗ p<0.001, ∗∗∗∗ p<0.0001, ns, non-significant. All replicate experiments were performed at least three times. See also Figure S2.

To provide further confirmation that the HiBiT tag is functionally linked to BRCA1 protein levels, we hypothesized that specific RNAi knockdown of *BRCA1* transcript levels would also reduce HiBiT luminescence detection. Indeed, we were able to effectively knockdown the BRCA1 protein level in HEK293T BRCA1-HiBiT reporter cells (Figure 2A), concomitant with HiBiT luminescence (Figure 2B). We also examined responses to non-selective modulators of BRCA1 protein levels to mimic potential screen hits. For instance, BRCA1 has been reported to undergo ubiquitin-mediated proteasomal degradation.^30^^,^^31^^,^^32^^,^^33^ Accordingly, treatment with the proteasome inhibitor MG132 led to the accumulation of BRCA1 protein in parental and targeted reporter clones (Figure 2C) and significantly increased HiBiT luminescence levels (Figure 2D). Next, to compare the kinetics of BRCA1 protein degradation with luminescence loss, cells were treated with the translational inhibitor cycloheximide (CHX). CHX reduced BRCA1 protein levels (Figure 2E) and HiBiT luminescence (Figure 2F) after 12 h of treatment. These findings indicate that BRCA1 protein level changes can be sensitively measured through HiBiT luminescence. Comparable outcomes were obtained in HeLa BRCA1-HiBiT reporter cells (Figures S2B–S2D), indicating the creation of reporter cells capable of sensitively measuring BRCA1 levels.

### Pilot screen identifies known epigenetic modulators of breast cancer susceptibility gene 1 expression

**Figure 3:**
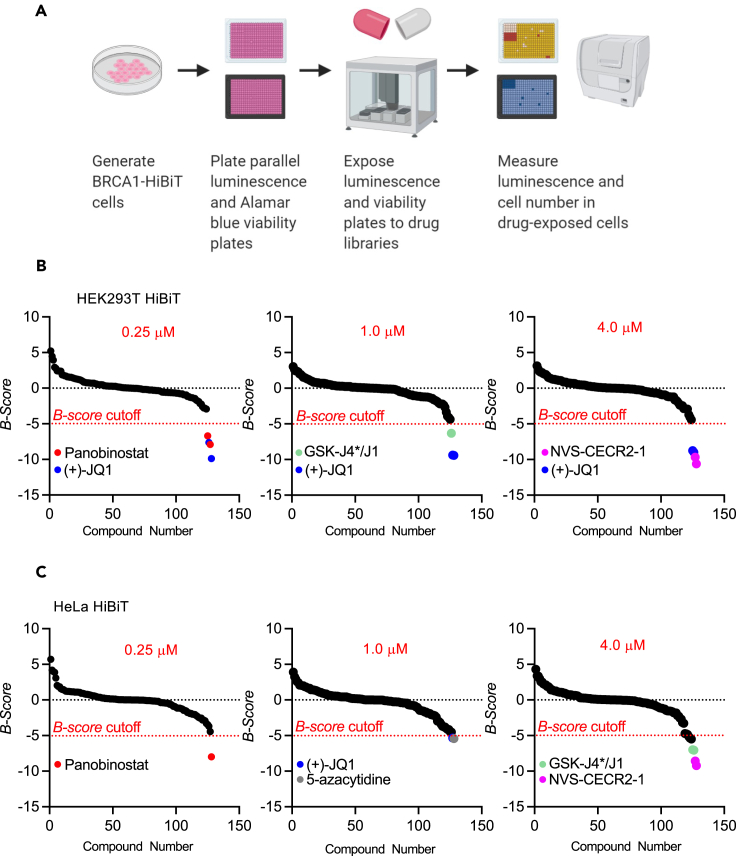
BRCA1-HiBiT reporter cell epigenetic probe library pilot screen (A) Workflow of screening protocol using HEK293T BRCA1-HiBiT cells with parallel luminescence and Alamar blue cell viability. (B) Ranked order of B-scores of HEK293T G7 cells treated with one of the 64 epigenetic-modifying compounds at 3 different concentrations (0.25, 1, and 4μM). (C) Ranked order of B-score of HeLa F11 cells treated with one of the 64 epigenetic-modifying compounds at 3 concentrations (0.25, 1, and 4μM).

To evaluate the efficacy of using BRCA1-HiBiT cell models in high-throughput drug screens, we performed a pilot screen with the Structural Genomics Consortium (SGC) Epigenetics Probes Collection which consists of 64 well-characterized epigenetic-modifying probe compounds (https://www.thesgc.org/chemical-probes). For this pilot screen, 3.5 x 10^3^ cells were seeded per well in a 384-well dish and treated with 0.25 μM, 1 μM, and 4 μM of each probe for 24 h. Simultaneous luminescence and viability assays were performed to identify toxic probes (Figure 3A). The pilot screens performed in both HEK293T and HeLa BRCA1-HiBiT reporter lines identified several probes that resulted in reduced HiBiT luminescence. In HEK293T BRCA1-HiBiT cells, we identified (+)-JQ1, GSK-J4∗/J1, NVS-CECR2-1, and panobinostat as candidate BRCA1 downregulators (Figure 3B; Table 1). In HeLa BRCA1-HiBiT cells, we also identified (+)-JQ1, GSK-J4∗/J1, NVS-CECR2-1, and panobinostat in addition to 5-azacytidine (Figure 3C; Table 2). B-score was used for hit selection in high-throughput screens as it is a measure of relative potency, similar to *Z* score, that is adjusted for positional effects.^34^^,^^35^ While we expected to find some shared hits between the HEK293T and HeLa reporter line screens, the extensive degree of overlap greatly bolstered our confidence that the two reporter lines were functioning similarly. The identification of (+)-JQ1 and panobinostat in this pilot was of specific interest, as other studies have previously identified these agents as BRCA1 downregulators. Previous studies showed that (+)-JQ1^36^^,^^37^^,^^38^ and panobinostat^39^^,^^40^^,^^41^^,^^42^ can reduce BRCA1 protein expression and sensitize cancer cells to PARPi. These results validated our screening approach and confirmed that it is capable of identifying compounds proven to downregulate BRCA1.

**Table 1:** HEK293T BRCA1-HiBiT pilot screen hits

Compound	Info	% Activity	Treatment	B Score
NVS-CECR2-1	CECR2, histone acetyl-lysine reader	3.3139834	4μM	−10.6465338
NVS-CECR2-1	CECR2, histone acetyl-lysine reader	18.224495	4μM	−9.75030982
(+)-JQ1	binds competitively to acetyl-lysine recognition motifs, or bromodomains	36.521417	4μM	−8.86488395
(+)-JQ1	binds competitively to acetyl-lysine recognition motifs, or bromodomains	42.779197	4μM	−9.01949310
GSK-J4∗/J1	Inhibition of histone H3K27 demethylase	45.850641	1μM	−6.83328481
(+)-JQ1	binds competitively to acetyl-lysine recognition motifs, or bromodomains	43.502488	1μM	−9.87129968
(+)-JQ1	binds competitively to acetyl-lysine recognition motifs, or bromodomains	47.795857	1μM	−9.86547759
LBH-589, Panobinostat	Panobinostat (LBH589) is a novel broad-spectrum HDAC inhibitor with an IC50 of 5 nM. Phase 3	65.241016	0.25μM	−8.92461144
LBH-589, Panobinostat	Panobinostat (LBH589) is a novel broad-spectrum HDAC inhibitor with an IC50 of 5 nM. Phase 3	73.299228	0.25μM	−7.97606728
(+)-JQ1	binds competitively to acetyl-lysine recognition motifs, or bromodomains	66.130723	0.25μM	−8.77119034
(+)-JQ1	binds competitively to acetyl-lysine recognition motifs, or bromodomains	64.338597	0.25μM	−11.1345986

**Table 2:** HeLa BRCA1-HiBiT pilot screen hits

Compound	Info	% Activity	Treatment	BScore
GSK-J4∗/J1	Inhibition of histone H3K27 demethylase	10.76938	4μM	−6.98975
GSK-J4∗/J1	Inhibition of histone H3K27 demethylase	8.236465	4μM	−7.05242
NVS-CECR2-1	CECR2, histone acetyl-lysine reader	1.46524	4μM	−8.57839
NVS-CECR2-1	CECR2, histone acetyl-lysine reader	1.544393	4μM	−9.22119
5-azacytidine	Azacytidine is a nucleoside analogue of cytidine that specifically inhibits DNA methylation by trapping DNA methyltransferases	77.97886	1μM	−5.43611
(+)-JQ1	binds competitively to acetyl-lysine recognition motifs, or bromodomains	63.77522	1μM	−5.34616
A-485	Histone Acetyltransferase Inhibitor	132.6749	0.25μM	5.699724
LBH-589, Panobinostat	Panobinostat (LBH589) is a novel broad-spectrum HDAC inhibitor with an IC50 of 5 nM. Phase 3	68.50157	0.25μM	−7.98186

We next performed the validation of (+)-JQ1, GSK-J4∗/J1, NVS-CECR2-1, and panobinostat to identify the most potent and reproducible BRCA1 downregulating molecules. The bromodomain inhibitor drug (+)-JQ1 significantly decreased BRCA1-HiBiT luminescence in HEK293T and HeLa reporter cells in a dose-dependent manner, in contrast to the inactive enantiomer (−)-JQ1 (Figures S3A–S3C and S3E). Importantly, neither (+)-JQ1 nor (−)-JQ1 significantly affected HEK293T nor HeLa cell viability at the 24-h time point of exposure (Figures S3D–S3F and S3G). Panobinostat also decreased BRCA1 luminescence in a dose-dependent manner in HEK293T and HeLa cells (Figures S4A and S4B) without any significant effects on cell viability at 24 h of exposure (Figure S4C). Although the compounds GSK-J4∗/J1 and NVS-CECR2-1 reduced BRCA1-HiBiT luminescence at certain doses in HEK293T cells (Figures S4D and S4E), GSK-J4∗/J1 and NVS-CECR2-1 were excluded from further study because they also reduced HEK293T cell viability in validation experiments (Figures S4F and S4G). Neither GSK-J4∗/J1 nor NVS-CECR2-1 reduced BRCA1 mRNA expression in MDA-MB-231 cells at the 4μM dose (Figure S4H and S4I).

### Validation of downregulating drug hits (+)-JQ1 and panobinostat in breast cancer cells

**Figure 4:**
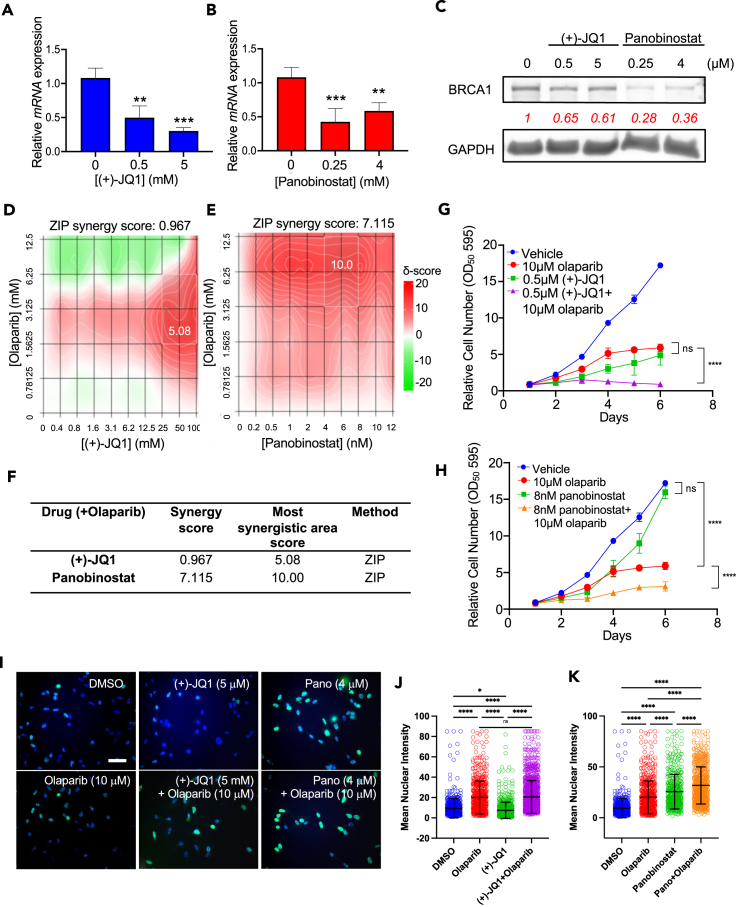
Validation of epigenetic probe library pilot screen candidates BRCA1 mRNA expression was measured by qRT-PCR after (A) (+)-JQ1 and (B) panobinostat treatment. Data are represented as mean ± SEM. ∗ p<0.05, ∗∗ p<0.01, ∗∗∗ p<0.001, ∗∗∗∗ p<0.0001, ns, non-significant. (C) Western blot demonstrating BRCA1 levels after treatment with (+)-JQ1 and panobinostat. Combination matrix heat maps of synergistic doses of (D) (+)-JQ1 and (E) panobinostat with olaparib. (F) Quantification of synergy between (+)-JQ1 or panobinostat with olaparib in MDA-MB-231 cells. Growth curves demonstrating combined drug effects between (G) (+)-JQ1 and (H) panobinostat and olaparib in MDA-MB-231 cells. Relative cell number for each timepoint is represented as mean ± SEM. ∗ p<0.05, ∗∗ p<0.01, ∗∗∗ p<0.001, ∗∗∗∗ p<0.0001, ns, non-significant. (I) gH2A.X immunofluorescence micrographs of MDA-MB-231 cells treated with (+)-JQ1 and panobinostat with or without olaparib. Scale bar: 100 μm. Quantitation of gH2A.X immunofluorescence intensity in MDA-MB-231 cells treated with (J) (+)-JQ1 with or without olaparib or (K) panobinostat with or without olaparib. ∗ p<0.05, ∗∗ p<0.01, ∗∗∗ p<0.001, ∗∗∗∗ p<0.0001, ns, non-significant.All replicate experiments were performed at least three times. See also and Figures S3–S5.

We next examined the capacity of the validated drugs to reduce BRCA1 expression in MDA-MB-231 cells, a triple-negative breast tumor cell line that is representative of BRCA1-expressing basal-like breast cancer. We observed that both (+)-JQ1 and panobinostat reduced *BRCA1* mRNA and protein expression in MDA-MB-231 cells in a dose-dependent manner (Figures 4A–4C). (+)-JQ1 and panobinostat were assessed for their capacity to induce synthetic lethality in combination with PARPi.

To determine the most effective doses for evaluating synthetic lethality, we examined the combined effects of various concentrations of (+)-JQ1 or panobinostat with olaparib on cell viability using the Synergy Finder tool and the Zero Interaction Potency (ZIP) method for quantification. MDA-MB-231 cells were treated in a 10 x 6 matrix of increasing (+)-JQ1 + olaparib dose combinations, or increasing panobinostat + olaparib dose combinations, and cell viability was measured (Figures S5A and S5B). In line with previous studies, we employed a synergy score threshold of 5 to identify synergy.^43^^,^^44^ Using the ZIP method, we generated synergy heatmaps **(**Figures 4D and 4E) where we observed that the overall (+)-JQ1 + olaparib synergy score was 0.97, however, the most synergistic combination had a score of 5.08 (Figures 4D and 4F). This suggests that (+)-JQ1 is not broadly synergistic with olaparib; however, the dose combination of 50μM (+)-JQ1 and 3.125μM olaparib demonstrates synergy. On the other hand, panobinostat + olaparib demonstrated an overall synergy score of 7.12, with maximal synergy 10.00 (Figures 4E and 4F). Thus, panobinostat is broadly synergistic with olaparib, and the dose combination of 6nM panobinostat and 6.25μM olaparib offers the greatest synergy.

Next, we performed growth curves with MDA-MB-231 cells treated with (+)-JQ1 (0.5 μM) or panobinostat (8 nM) alone or in combination with olaparib (10 μM). We observed that olaparib decreased cell growth alone. (+)-JQ1 also resulted in decreased cell growth at 0.5μM; however, co-treatment with (+)-JQ1 and olaparib reduced growth beyond the single doses (Figure 4G). On the other hand, 8nM panobinostat alone was well tolerated by MDA-MB-231 cells, but when combined with olaparib, panobinostat significantly reduced growth compared to olaparib alone (Figure 4H). These findings indicate that both (+)-JQ1 and panobinostat can be combined with olaparib to reduce the growth of MDA-MB-231 breast cancer cells.

To further assess the impact of BRCA1 expression loss mediated by (+)-JQ1 or panobinostat, we evaluated gH2A.X expression as a marker for DNA damage. In these experiments, MDA-MB-231 cells were treated with 5 μM (+)-JQ1 or 4 μM panobinostat with or without 10μM olaparib for 24 h. gH2A.X levels were quantitated using both microscopy (Figures 4I–4K) and flow cytometry (Figures S5C and S5D) with consistent results obtained from both methods. Notably, (+)-JQ1 alone did not induce any increase in DNA damage and failed to enhance DNA damage when combined with olaparib. In contrast, panobinostat alone elevated DNA damage levels and significantly increased DNA damage when combined with olaparib (Figure 4K). Together, these data indicate that both (+)-JQ1 and panobinostat reduce BRCA1 protein expression and can synergize in reducing the growth of MDA-MB-231 cells; however, panobinostat induces higher levels of DNA damage and accordingly appears to cooperate more effectively with olaparib.

### Screening the medical collection libraries with breast cancer susceptibility gene -HiBiT reporter cells

**Figure 5:**
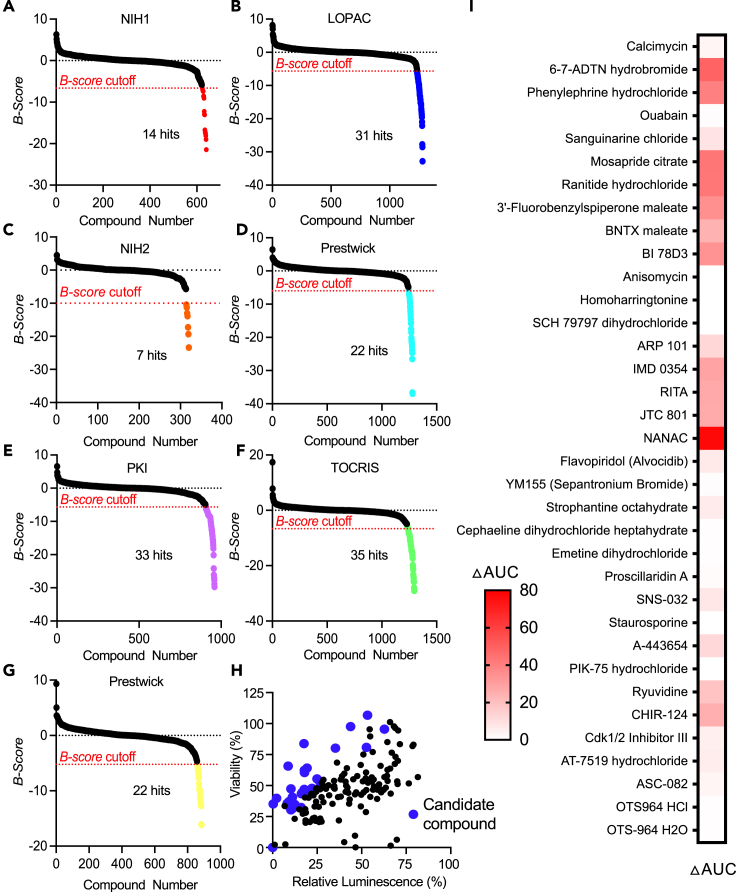
BRCA1-HiBiT reporter Medical Collection Screens Ranked B-score hits from the HEK293T BRCA1-HiBiT Medical Collection screen are as follows (A) NIH1, (B) LOPAC, (C) NIH2, (D) Prestwick, (E) PKI, (F) TOCRIS, and (G) Prestwick. (H) Activity vs. viability analysis was performed using the data from the luminescence screens and the viability screens. Blue dots indicate compounds that met a 2:1 ratio of viability: luminescence cut-off. (I) Combination screen was performed in MDA-MB-231 cells using 4 doses of each probe drug and 1 dose of olaparib. Dose-response curves were generated for each condition and area-under-the-curve (AUC) was calculated for each compound from (h). See also Figure S6 and Table S1.

After achieving favourable results from our pilot screening, we proceeded with a high-content screen aimed at identifying BRCA1 downregulating compounds that can induce synthetic lethality with PARPi. To conduct this expanded high throughput screen, we utilized the Medical Collection, which consists of compound libraries containing substances with well-defined mechanisms of action targeting specific molecular entities, cellular signaling pathways, or biological processes. The Medical Collection encompasses nearly 6000 compounds organized into seven sub-libraries: the TOCRIS library, the LOPAC-Sigma library, the PKI library, the Prestwick Chemical collection, the Selleck Chemical collection, and the NIH1 and NIH2 drug libraries (Table 3). For the Medical Collection screen, HEK293T BRCA1-HiBiT reporter cells were treated with DMSO as a vehicle control, MG132 as a positive control for altered BRCA1 expression, and the library for 24 h. The NIH1, NIH2, LOPAC, and TOCRIS libraries were screened at 4μM and the Selleck, Prestwick, and PKI libraries were screened at 1μM due to known toxicity. The screen identified a total of 163 unique compounds that strongly decreased luminescence (Figures 5A–5G; Table S1).

**Table 3:** Medical Collection Screen sub-libraries

Library	Size	Composition
TOCRIS	1296	Biologically active compounds
LOPAC - Sigma	1267	Chemicals with proven biological activity
PKI/OICR	720	Kinase inhibition sublibrary of OICR collection
Prestwick Chemical	1120	85% off-patent, FDA approved drugs
Selleck Chemical	885	Kinase inhibitors with known targets
NIH1	446	Drugs tested in phase I, II, and III clinical trials
NIH2	281	Drugs tested in phase I, II, and III clinical trials

Given the large number of BRCA1-downregulating hits identified in the screen, we performed a series of sub-screens to identify optimal candidates. First, an independent viability screen was performed on the 163 BRCA1-downregulating compounds to exclude those compounds with cytotoxic effects. Viability was assessed in HEK293T BRCA1-HiBit cells 24 h post-drug treatment using doses identical to those used in the full screen. Compounds with a favourable viability profile were identified by calculating a luminescence/viability ratio for each compound (Figure 5H). This screen identified 35 compounds with specific effects on HiBiT luminescence and minimal cytotoxicity (Table 4).

**Table 4:** HEK293T BRCA1-HiBiT Medical Collection screen hits

#	Library	Compound	%Activity	B score	%Viability	*Z* score	Ratio
20	LOPAC	Ouabain	9.296	−27.727	37.101	−2.215	3.991
30	LOPAC	Sanguinarine chloride	12.448	−28.528	34.774	−2.297	2.794
5	LOPAC	Calcimycin	22.102	−20.998	47.803	−1.838	2.163
18	LOPAC	Phenylephrine hydrochloride	53.312	−15.238	106.761	0.238	2.003
7	LOPAC	6-7-ADTN hydrobromide	62.816	−8.957	95.505	−0.158	1.520
33	NIH1	MOSAPRIDE CITRATE	43.757	−12.994	97.569	−0.086	2.230
52	NIH2	SAM002264645	8.002	−23.408	34.318	−2.313	4.289
65	PKI	Ryuvidine	8.751	−18.128	65.675	−1.209	7.505
85	PKI	OTS-964 H2O	16.188	−29.742	43.383	−1.994	2.680
82	PKI	OTS964 HCl	16.643	−27.016	44.423	−1.957	2.669
61	PKI	Staurosporine, AM-2282	17.751	−14.561	46.879	−1.870	2.641
76	PKI	ASC-082, APY 82	12.864	−9.734	33.319	−2.348	2.590
71	PKI	Cdk1/2 Inhibitor III	18.807	−8.394	43.932	−1.974	2.336
60	PKI	SNS-032, BMS387032, BMS-387032	24.980	−14.258	55.361	−1.572	2.216
64	PKI	PIK-75 hydrochloride	19.440	−16.433	42.054	−2.040	2.163
72	PKI	AT-7519 hydrochloride, AT7519 hydrochloride	21.706	−8.657	46.097	−1.898	2.124
63	PKI	A-443654, A-654	15.002	−15.575	30.826	−2.436	2.055
67	PKI	CHIR-124	26.495	−7.074	53.120	−1.651	2.005
96	Prestwick	Strophantine octahydrate	10.304	−21.967	47.382	−1.853	4.598
97	Prestwick	Cephaeline dihydrochloride heptahydrate	8.247	−23.836	35.650	−2.266	4.323
98	Prestwick	Emetine dihydrochloride	10.629	−26.539	35.341	−2.277	3.325
107	Prestwick	Proscillaridin A	16.000	−36.979	32.304	−2.384	2.019
114	Selleck	YM155 (Sepantronium Bromide)	0.362	−12.689	35.154	−2.283	97.171
113	Selleck	Flavopiridol (Alvocidib)	21.626	−10.650	46.127	−1.897	2.133
161	TOCRIS	JTC 801	2.186	−14.452	39.749	−2.122	18.183
162	TOCRIS	N-Acetyl-N-acetoxy-4-chlorobenzenesulfonamide	17.603	−14.777	83.850	−0.569	4.764
144	TOCRIS	Homoharringtonine	9.680	−29.073	35.916	−2.256	3.710
159	TOCRIS	RITA	11.118	−12.797	40.557	−2.093	3.648
141	TOCRIS	BI 78D3	17.807	−23.709	62.272	−1.328	3.497
135	TOCRIS	BNTX maleate	17.879	−20.773	60.462	−1.392	3.382
158	TOCRIS	IMD 0354	19.343	−12.431	64.086	−1.265	3.313
154	TOCRIS	SCH 79797 dihydrochloride	10.249	−29.078	30.315	−2.454	2.958
143	TOCRIS	Anisomycin	11.168	−27.901	31.554	−2.410	2.826
155	TOCRIS	ARP 101	35.268	−8.938	80.162	−0.699	2.273
134	TOCRIS	3′-Fluorobenzylspiperone maleate	52.717	−11.014	80.748	−0.678	1.532

Next, to identify BRCA1-downregulating compounds that cooperate with PARPi in breast cancer cells, the 35 candidate drugs were tested for their capacity to sensitize MDA-MB-231 cells to olaparib. For this sub-screen, a single dose of olaparib (5μM) was combined with 4 doses of each candidate compound for 72 h, after which viability was assessed. 5μM olaparib was chosen as it was previously found to represent an EC_10_ dose at 72 h in MDA-MB-231 cells, and therefore any drug that reduced growth greater than 10% when dosed with olaparib was determined to combine with olaparib. Dose-response curves were generated for each drug alone or in combination with olaparib, and differential area-under-the-curve (ΔAUC) was calculated for each treatment combination. ΔAUC, in this context, represents the difference in viability in cells treated with monotherapy or combination. Nineteen of the 35 compounds demonstrated significantly different viability compared to each compound as a single agent (Figure 5I; Figure S6) and seven out of those 19 candidate compounds were found to reduce viability greater than 10% in combination with olaparib.

**Figure 6:**
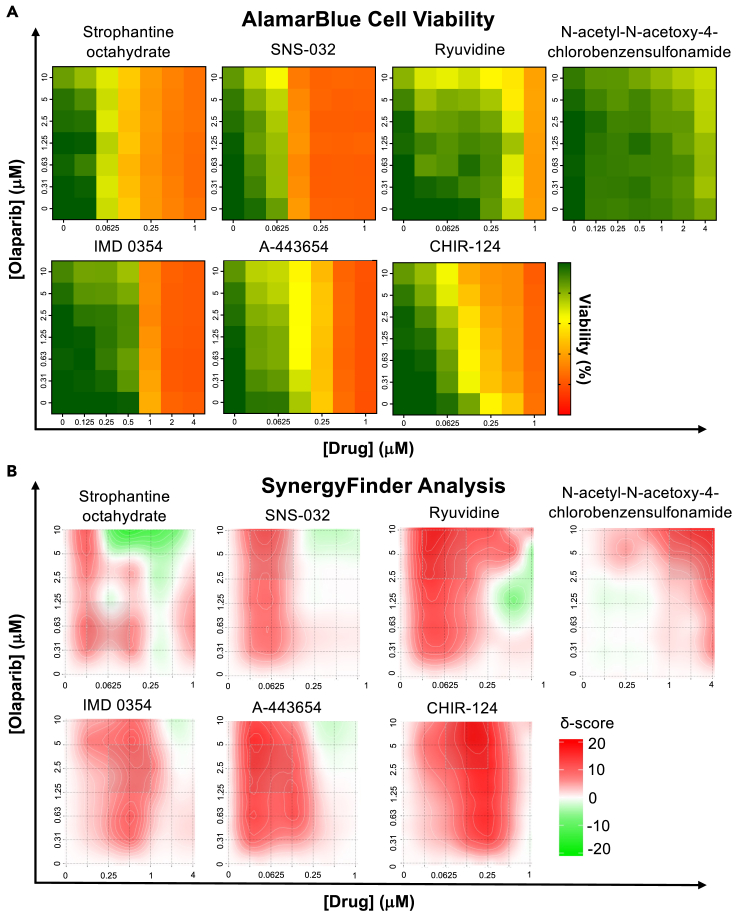
Viability and synergy assessment of screen candidates (A) Dose-response matrices were generated for each of the top seven downregulating drugs in combination with olaparib. (B) Synergy analysis was performed using the zero-interaction potency (ZIP) synergy scoring method. Olaparib was used at doses of 0, 0.3125, 0.625, 1.25, 2.5, 5, and 10 μM. Candidate drugs SO, SNS-032, ryuvidine, A-443654, and CHIR-124 were used at doses of 0, 0.03125, 0.0625, 0.125, 0.25, 0.5, and 1 μM. Candidate drugs N-acetyl-N-acetoxy-4-chlorobenzensulfonamide and IMD-0354 were used at doses of 0, 0.125, 0.25, 0.5, 1, 2, and 4 μM. Cell viability was measured by Alamar Blue and results used to generate heat maps and synergy maps. See also Figure S6.

The seven compounds identified in this screening included IMD-0354, N-acetyl-N-acetoxy chlorobenzenesulfonamide (NANAC), strophantine octahydrate (SO), SNS-032, A-443654, ryuvidine, and CHIR-124 (Figure S7). These selected candidates underwent subsequent screening to evaluate synergy with olaparib across various dosage combinations. For this, MDA-MB-231 cells underwent treatment with 49 combinations of drugs with olaparib (arranged in a 7x7 matrix) for 72 h. Cell viability and synergy were assessed using the Synergy Finder tool employing the ZIP method as before to determine both overall and maximum synergy scores (Figures 6A and 6B). Among candidate compounds, SO did not demonstrate synergy with olaparib at any dose combination (Table 5). By contrast, SNS-032, ryuvidine, NANAC, and IMD-0354 demonstrated overall synergy scores below the synergy threshold of 5, however, each of these compounds did achieve synergy with olaparib at some specific dose combinations (Table 5). A-443654 and CHIR-124 demonstrated the highest overall synergy scores of 5.49 and 11.86, respectively, suggesting these compounds display broad synergy with olaparib across a range of doses and even more potent effects on cell viability at optimal doses (Table 5).

**Table 5:** MDA-MB-231 olaparib synergy screen hits

Drug (+Olaparib)	Synergy score	Most synergistic area score	Method
Strophantine octahydrate	0.40	2.18	ZIP
SNS-032	2.86	6.41	ZIP
Ryuvidine	2.94	6.52	ZIP
N-acetyl-N-acetoxy-4-chlorobenzenesulfonamide	3.69	10.69	ZIP
IMD 0354	3.91	6.92	ZIP
A-443654	5.45	11.79	ZIP
CHIR-124	11.86	21.58	ZIP

### Candidate compounds downregulate breast cancer susceptibility gene protein expression in MDA-MB-231 cells

**Figure 7:**
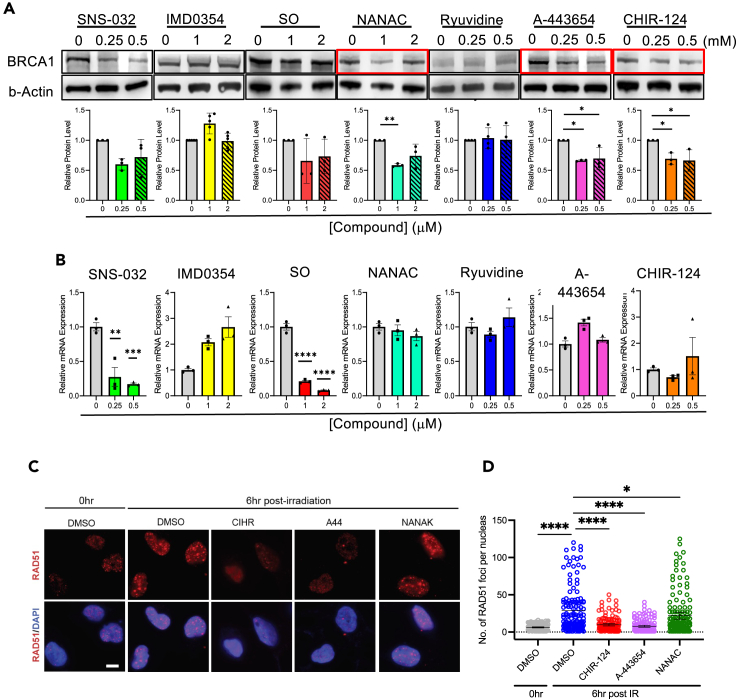
Validation of Medical Library screen candidates (A) BRCA1 Western blots with quantitation in MDA-MB-231 cells treated with depicted does of each of the top 7 candidate compounds. Data are represented as mean ± SEM. ∗ p<0.05, ∗∗ p<0.01, ∗∗∗ p<0.001, ∗∗∗∗ p<0.0001, ns, non-significant. (B) *BRCA1* qRT-PCR of MDA-MB-231 cells treated with depicted does of each of the top 7 candidate compounds. Data are represented as mean ± SEM. ∗ p<0.05, ∗∗ p<0.01, ∗∗∗ p<0.001, ∗∗∗∗ p<0.0001, ns, non-significant. (C) Rad51 immunofluorescence in MDA-MB-231 cells subjected to 8 Gy of IR in the presence of NANAC, A-443654, and CHIR-124 Scale bar: 20 μm. (D) Quantitation of Rad51 foci. All replicate experiments were performed at least three times and representative images shown. ∗ p<0.05, ∗∗ p<0.01, ∗∗∗ p<0.001, ∗∗∗∗ p<0.0001, ns, non-significant. See also Figures S8 and S9.

To validate the ability of candidate compounds identified in our screens to lower endogenous BRCA1 expression, we conducted both Western blot and qRT-PCR in MDA-MB-231 cells treated with candidate compounds. Among the 7 top candidate compounds, A-443654 and CHIR-124 demonstrated the consistent downregulation of BRCA1 protein levels, and NANAC was able to do so only at 1μM (Figure 7A). Comparable downregulation for these compounds was also observed in HEK293T cells (Figure S8A). Despite trends of downregulation with SNS-032 and SO, they did not reach significance in MDA-MB-231 cells (Figure 7A). qRT-PCR showed that *BRCA1* transcript levels were most significantly downregulated in cells treated with SNS-032 and SO suggesting that these compounds likely have effects on *BRCA1* transcript (Figure 7B). None of the other compounds were able to reduce *BRCA1* transcript levels (Figure 7B). Overall, these data suggest that A-443654, CHIR-124, and NANAC lead to BRCA1 downregulation, most likely through post-transcriptional mechanisms.

To assess how decreasing the levels of BRCA1 protein affects its direct cellular functions, we examined the formation of Rad51 foci in MDA-MB-231 when exposed to the BRCA1 downregulating compounds NANAC, A-443654, and CHIR-124. Specifically, cells exposed to NANAC, A-443654, and CHIR-124 were treated with or without 8 Gy of gamma irradiation (gIR) to induce DNA damage. In unirradiated cells, Rad51 foci formation was minimal, and no notable changes in the number of Rad51 foci were observed (Figures 7C and 7D). However, following DNA damage induced by gIR, there was a significant increase in Rad51 foci formation (Figures 7C and 7D). Importantly, treating cells with A-443654, CHIR-124, or NANAC significantly decreased the number of Rad51 foci 6 h after gIR (Figures 7C and 7D). These data indicate that the downregulation of BRCA1 with A-443654, CHIR-124, or NANAC also decreases BRCA1 function in DDR.

We also examined the effects of all candidate compounds on other proteins involved in HR by Western blot and qRT-PCR. Among the validated BRCA1 downregulating compounds, we observed that NANAC treatment reduced BRCA2 and Rad51 protein levels, only at the 1μM dose, but not at the higher dose, consistent with observations for BRCA1 (Figures S8B–S8D). A-443654 also reduced BRCA2 and Rad51 protein levels at the higher dose (Figures S8B–S8D). On the other hand, CHIR-124 did not lead to significant changes in other HR protein levels, suggesting a selective effect on BRCA1 protein (Figures S8B–S8D). To identify if all seven candidate drugs are acting in transcription or post-transcriptionally on other HR proteins, we also evaluated transcript level changes (Figures S9A–S9C). As with *BRCA1*, we observed that SNS-032 and SO also strongly downregulated the expression of *BRCA2, PALB2,* and *Rad51* mRNA expression, however, they did not significantly impact protein expression (Figures S8A–S8C and S9A–S9C). A-443654, NANAC, and CHIR-124 did not significantly downregulate *BRCA2, PALB2,* or *Rad51* mRNA expression (Figures S9A–S9D), suggesting that these compounds may alter protein level post-transcriptionally.

### Breast cancer susceptibility gene downregulating compounds sensitize breast cancer cells to the effects of olaparib

**Figure 8:**
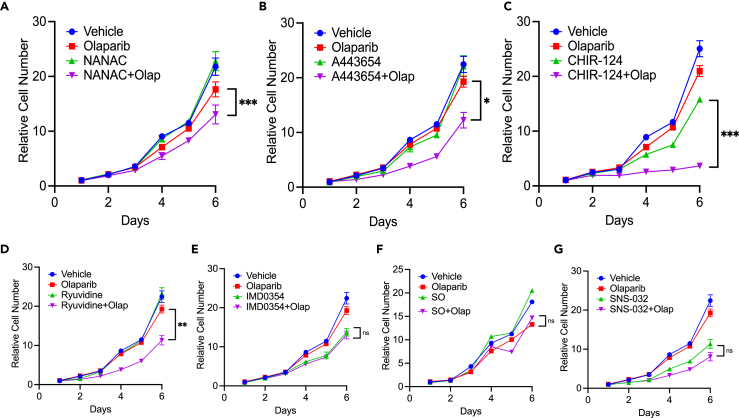
NANAC, A-443654, and CHIR-124 combine with olaparib to reduce breast cancer growth Growth curve analysis of (A) NANAC (0.25μM); (B) A-443654 (0.0625μM); and (C) CHIR-124 (0.05μM); (D) ryuvidine (0.5μM); (E) IMD-0354 (0.25μM); (F) SO (0.025μM); and (G) SNS-032 (0.0625μM]) with or without 2.5μM olaparib. Relative cell number for each timepoint is represented as mean ± SEM. ∗ p<0.05, ∗∗ p<0.01, ∗∗∗ p<0.001, ∗∗∗∗ p<0.0001, ns, non-significant. All replicate experiments were performed at least three times.

Finally, we investigated the impact of each of the seven candidate compounds on cell growth in the MDA-MB-231 cell line, both with and without olaparib. Beginning with the three validated BRCA1 downregulating compounds — NANAC, A-443654, and CHIR-124 — we found that all of them could synergize with olaparib, resulting in a reduction in MDA-MB-231 cell growth (Figures 8A–8C). Interestingly, despite minimal effects on reducing BRCA1 levels, ryuvidine also demonstrated synergy with olaparib in growth experiments, suggesting a potential BRCA1-independent mechanism of synergy with olaparib. However, IMD-0354, SNS-032, and SO did not display significant synergy with olaparib (Figures 8D–8G), indicating their limited efficacy in this context.

## Discussion

PARPi have demonstrated excellent potential in clinical settings, especially when administered to patients with *BRCA1* and *BRCA2*-associated breast and ovarian cancer.^8^^,^^10^^,^^13^^,^^45^ Similarly, mutations in other HR genes which confer BRCA-like BRCAness phenotypes also demonstrate synthetic lethality when treated with PARPi.^6^ Nevertheless, these mutations are found in a limited proportion of cancers, restricting the therapeutic applicability of PARPi. Therefore, identifying strategies to expand the use of PARPi could present great benefits for diverse cancer types. Recent studies have demonstrated that small molecule drugs targeting diverse molecular pathways associated with HR can trigger therapeutic BRCAness.^41^^,^^46^ Our objective was to identify compounds that reduce BRCA1 protein levels, as a means of pharmacologically inducing synthetic lethality upon combination with PARPi. The discovery of such agents could expand the applicability and efficacy of PARPi strategies in the treatment of wild-type *BRCA1* tumors. Additionally, these agents could be used for the treatment of tumors that have developed PARPi-resistance after treatment, especially those that harbor reversion mutations that restore BRCA1 function.^9^^,^^45^^,^^47^

The success of our approach hinges on an effective means to identify BRCA1-suppressing compounds in a high throughput manner. CRISPR/Cas9 editing facilitated the generation of BRCA1-HiBiT reporter cell lines that permit the sensitive detection of changes in BRCA1 protein levels. Importantly, our cell models address limitations of existing BRCA1 reporter models, including BRCA1 overexpression, cell cycle inhibition, and apoptosis.^48^^,^^49^^,^^50^ Moreover, since HiBiT is a tiny tag engineered into the *BRCA1* locus, we anticipate that it will result in minimal interference with transcriptional, epigenetic, and translational regulatory processes as compared to models with larger fluorescent protein tags. In sum, the luminescence-based HiBiT system is particularly valuable for high-throughput measurements of BRCA1 levels since it is exquisitely sensitive and can be employed in a high-throughput format.

In our preliminary screen designed to evaluate the feasibility of using our newly generated reporter cell lines, we identified two agents, (+)-JQ1 and panobinostat, which exhibited a capacity to decrease BRCA1 expression in both HEK293T and HeLa reporter lines. In MDA-MB-231 triple-negative breast cancer cells, both compounds reduced BRCA1 levels while also increasing sensitivity to olaparib. Importantly, (+)-JQ1 has been established as a down-regulator of BRCA1 in various cancer cell types^36^^,^^38^ and was demonstrated to cooperate with PARPi to exacerbate cytotoxicity in pancreatic ductal and ovarian carcinoma.^36^^,^^46^ Similarly, panobinostat was reported to enhance the sensitivity of HR-competent ovarian carcinoma cells to PARPi.^39^^,^^42^ Therefore, our identification of (+)-JQ1 and panobinostat through completely independent screening methods validates these agents as bona-fide BRCA1 downregulators and underscores the value of our BRCA1-HiBiT reporter cells in identifying drugs that can synergize with PARPi.

In high-content screens, we identified seven previously unreported compounds that significantly downregulated BRCA1-HiBiT and exhibited synergy with olaparib. These agents include SO, SNS-032, ryuvidine, NANAC, IMD-0354, A-443654, and CHIR-124. Although we could not identify a common molecular function among these compounds, our analysis of the known mechanisms of action of these compounds identified putative links to BRCA1 expression. For example, SO is a cardiac glycoside most commonly referred to as ouabain^51^ and has been demonstrated to influence DNA damage repair protein Fanconi anemia group D2 protein (FANCD2), a protein that directly interacts with BRCA1.^52^ Notably, ouabain has been studied as an anti-cancer therapeutic in prostate, breast, and lung cancers, as well as osteosarcoma.^51^^,^^53^^,^^54^^,^^55^^,^^56^ SNS-032 is a CDK inhibitor targeting CDK2, 7 and 9^57^^,^^58^ that exerts several anti-cancer effects, including reducing cell growth, inducing apoptosis, and inhibiting angiogenesis and invasion in various cancer types, including esophageal cancer, AML, breast cancer, and multiple myeloma.^59^^,^^60^^,^^61^^,^^62^ Although there is no data to date for SNS-032, the CDK7 inhibitors THZ1 and THZ2 reduced the transcription of *BRCA1, BRCA2,* and *Rad51*.^63^ Ryuvidine has been shown to inhibit the function of the lysine-specific demethylase 5A (KDM5A) that demethylates H3K4me3.^64^^,^^65^ KDM5A is implicated in transcriptionally regulating genes involved in cell growth, differentiation and stemness, drug resistance, and metastasis.^65^ Ryuvidine has also been implicated in cell cycle control and DNA replication, as it reduces the expression of cell cycle division 7-related protein kinase (CDC7) and stimulates the DNA damage response.^66^ IMD-0354 functions as a specific inhibitor of the inhibitor of nuclear factor kappa-B kinase subunit β(IKKβ).^67^ BRCA1 and NF-kB complex together to mediate transcriptional activation and mediate DSB repair^68^^,^^69^ NF-κB consensus sequence binding sites have been discovered in the 5′ region of the BRCA1 promoter^70^^,^^71^ and, the inhibition of NF-kB by the IKKβ inhibitor BMS-345541 reduces BRCA1 expression along with other DNA repair genes, including BRCA2, Rad51, and FANCD2.^72^^,^^73^ These findings support a potential mechanism of IMD-0354 in reducing BRCA1 protein levels and inducing PARPi sensitivity.

Although SO, SNS-032, ryuvidine, and IMD-0354 were hits in the HiBiT screen, we were unable to confirm their ability to reduce BRCA1 protein levels in MDA-MB-231 cells. This could suggest these compounds exert tissue-specific effects on BRCA1 protein or mRNA levels and could warrant further study. On the other hand, NANAC, A-443654, and CHIR-124 demonstrated notable efficacy in suppressing both the expression and function of BRCA1 protein. When combined with olaparib, they exhibited a capacity to significantly diminish the proliferation of breast cancer cells. NANAC is a pro-drug of nitroxyl (HNO), which is a type of nitrogen oxide (NO) that exhibits vasorelaxant properties, stimulating vasodilation, muscle relaxation, and myocardial contractility modulation.^74^^,^^75^ NANAC has been shown to protect ischemic myocardium and has been investigated for its potential therapeutic applications in conditions such as heart failure and ischemic heart disease.^74^^,^^75^ NANAC has also been shown to inhibit aldehyde dehydrogenase enzymes, which are involved in the detoxification and clearance of aldehydes, such as those produced by alcohol metabolism and synthesizing amino acids, carbohydrates, and lipids and antagonize the neuroprotective effects of neuroprotective effects of PPARγ.^76^^,^^77^ NANAC has not been studied in cancer, nor has it been linked to BRCA1 in any studies.

A-443654 is an ATP-competitive small-molecular AKT inhibitor.^78^^,^^79^ It has been investigated in preclinical studies as a potential treatment for various types of cancer, including breast cancer. Interestingly, previous studies have illustrated that AKT inhibition can sensitize BRCA1^+/+^ tumors to PARPi.^80^^,^^81^ Indeed, activated AKT can increase the expression of BRCA1 and BARD1, and PI3K inhibitors that function upstream of AKT have been shown to decrease BRCA1 and BARD1 expression.^82^ These findings suggest that the activated PI3K/AKT pathway influences BRCA1 expression and thus the inhibition of AKT by A-443654 may reduce BRCA1 expression in a similar fashion.

In our studies, CHIR-124 demonstrated the highest synergy with olaparib in decreasing breast cancer cell viability. CHIR-124 is a potent quinolone ATP competitive inhibitor of Chk1, a kinase that plays multiple critical roles in the sensing and repair of DSBs in conjunction with BRCA1.^83^^,^^84^^,^^85^ *In vitro*, CHIR-124 also potently targets other kinases such as PDGFR (IC50, 6.6nM) and FLT3 (IC50, 5.8nM).^86^ CHIR-124 treatment synergizes with topoisomerase inhibitors,^83^ mTOR inhibition using rapamycin,^87^ HDAC inhibitors such as vorinostat, romidepsin, and entinostat,^88^ DNA-intercalating drugs,^89^ and radiation.^90^ This synergy results in decreased cancer cell proliferation, G2/M checkpoint arrest, and apoptosis. Other Chk1 inhibitors, such as MK-8776 and Lys2606368 synergize with PARPi in olaparib-resistant ovarian cancer^91^^,^^92^ and gastric cancer.^93^ Our findings suggest that CHIR-124 could offer similar benefits in both BRCA1-mutant and wild-type breast and ovarian cancers when combined with PARPi. CHIR-124 has been shown to synergize with gemcitabine in pancreatic cell culture,^94^ and an alternate Chk1 inhibitor, SRA737, is being tested in Phase I/II clinical trials in combination with gemcitabine in anogenital cancer, cervical cancer, ovarian cancer, rectal cancer, and non-small cell lung cancer.^95^ While the impact of CHIR-124 on BRCA1 expression remains unexplored, it is plausible that CHIR-124 synergizes with PARPi through two mechanisms: direct Chk1 inhibition and reduction in BRCA1 expression.

In sum, NANAC, A-443654, and CHIR-124 represent previously unknown agents for downregulating BRCA1 expression, which are largely unstudied in cancer treatment. Based on these findings, we conclude that our reporter cell models serve as effective tools for the identification of modifiers and downregulators of BRCA1 expression. These reporter lines are particularly valuable as they incorporate disparate pathways that encompass diverse networks converging on BRCA1 regulation, including transcription, mRNA regulation, translation, protein modification, and protein-protein interaction. Future investigations can assess whether tumor cells expressing mutant BRCA1 exhibit unique sensitivity to BRCA1 downregulating compounds. Ongoing research efforts are focused on further analysis of candidate agents, and exploration of a broader spectrum of compounds, including naturally derived product libraries.

### Limitations of the study

This study also has some limitations. The HEK293T and HeLa BRCA1-HiBiT reporter cells have not been validated for BRCA1 functionality and therefore may reflect changes in levels but not changes in protein structure or function. Further analysis of BRCA1 function in Rad51 foci formation could elucidate the efficacy of our reporter lines in this context. Additionally, we have shown that NANAC, A-443654, and CHIR-124 reduce BRCA1 protein expression post-transcriptionally, but we have not identified the specific mechanism of action for each compound. Further studies could explore the exact signaling cascades and protein interactions interrupted by each of these compounds that leads to the downregulation of BRCA1 protein level. Additionally, while these compounds reduce BRCA1 protein level *in vitro* in a triple-negative breast cancer cell model, further studies are necessary to evaluate the tissue-specificity of these compounds. Future experimentation in other *in vitro* models of breast cancer as well as *in vivo* mouse mammary models could determine the toxicity of these compounds, their utility in treating *BRCA1* wild-type tumors, and the value of these compounds in the treatment of PARPi-resistant tumors that have undergone reversion mutations.

## STAR★Methods

### Key resources table

**Table:** 

REAGENT or RESOURCE	SOURCE	IDENTIFIER
**Antibodies**		

BRCA1 (MS110)	Millipore Sigma	cat#: OP92; RRID:AB_2750876
BRCA2 (D9S6V)	Cell Signaling Technology	cat#: 10741; RRID:AB_2797730
PALB2 (E9R2W)	Cell Signaling Technology	cat#: 30253; RRID:AB_2895010
Rad51 (D4B10)	Cell Signaling Technology	cat#: 8875; RRID:AB_2721109
GAPDH (14C10)	Cell Signaling Technology	cat#: 2118; RRID:AB_561053
phospho-histone H2A.X (Ser139) (JW301)	Millipore Sigma	cat#: 16-202A; RRID:AB_568825
anti-mouse HRP	Kindle Biosciences	cat#: 1005; RRID:AB_2800463
anti-rabbit HRP	Kindle Biosciences	cat#: 1007; RRID:AB_2800464
Alexa Fluor 488 Goat anti-rabbit	Invitrogen	cat#: A31627; RRID:AB_3073868
Rad51 (ab176458)	Abcam	cat#: ab176458; RRID:AB_2665405
Alexa Fluor 594 Goat anti-rabbit	Invitrogen	cat#: A11012; RRID:AB_2534079
Alexa Fluor 488 Goat anti-rabbit	Invitrogen	cat#: A31627

**Chemicals, peptides, and recombinant proteins**		

MG132	Millipore	cat#: 474790
Cycloheximide	Cell Signaling Technologies	cat#: 2112
EnGen Spy Cas9 NLS Protein	New England Biolabs	cat#: M0646T

**Critical commercial assays**		

Nano-Glo HiBiT Lytic System	Promega	cat#: N3030
Nano-Glo HiBiT Blotting System	Promega	cat#: N2410
DharmaFECT 1 Transfection Reagent	Dharmacon	cat#: T-2001
Lipofectamine RNAiMax Transfection Reagent	Thermofisher	cat#: 13778075
QIAquick PCR Purification Kit	Qiagen	cat#: 28106
RNeasy Mini Kit	Qiagen	cat#: 74106
SuperScript IV VILO with ezDNase Master Mix Kit	Invitrogen	cat#: 11766050
TaqMan Fast Advanced Master Mix	Applied Biosystems	cat#: 4444556
Taqman mRNA assay: BRCA1	Applied Biosystems	cat#: Hs01556193_m1
Taqman mRNA assay: Rad51	Applied Biosystems	cat#: Hs00947967
Taqman mRNA assay: BRCA2	Applied Biosystems	cat#: Hs00609073
Taqman mRNA assay: PALB2	Applied Biosystems	cat#: Hs00954121
Taqman mRNA assay: Beta Actin	Applied Biosystems	cat#: Hs01060665_g1
Taqman mRNA assay: GAPDH	Applied Biosystems	cat#: Hs02786624_g1
Alamar Blue Cell Viability	Invitrogen	cat#: DAL1025
H2A.X Phosphorylation Assay Kit (Flow Cytometry)	Millipore Sigma	cat#: 17-344
Click-iT™ EdU Cell Proliferation Kit for Imaging, Alexa Fluor 488 dye	Invitrogen	cat#: C10337

**Experimental models: Cell lines**		

Human: HEK293T cell	American Type Cell Collection	cat#: CRL-3216
Human: HEK293T BRCA1-HiBiT	This paper	
Human: HeLa cell	American Type Cell Collection	cat#: CRM-CCL-2; RRID:CVCL_0030
Human: HeLa BRCA1-HiBiT	This paper	
Human: MDA-MB-231 cell	American Type Cell Collection	cat#: CRM-HTB-26
Human: MCF7 cell	American Type Cell Collection	cat#: HTB-22
Human: U2OS cell	American Type Cell Collection	cat#: HTB-96
Human: DU145 cell	American Type Cell Collection	cat#: HTB-81

**Oligonucleotides**		

ssODN: BRCA1-HiBiT HDR: TGCCAGGAGCTGGACACCTACCTGATACCCCA GATCCCCCACAGCCACTACGTCTCCGTGAGCG GCTGGCGGCTGTTCAAGAAGATTAGCTGACTG CAGCCAGCCACAGGTACAGAGCCACAGGACC CCAAGAATGAGCT	Integrated DNA Technologies	This paper
sgBRCA1: TACTGACTGCAGCCAGCCAC	Integrated DNA Technologies	This paper
Primer (BRCA1 F1): GCAATTGGGCAGATGTGTGA	Integrated DNA Technologies	This paper
Primer (BRCA1 R1): ACCCTTGCATAGCCAGAAGT	Integrated DNA Technologies	This paper
Primer (BRCA1 N-F1): GGCGGCTGTTCAAGAAGATT	Integrated DNA Technologies	This paper
Primer (BRCA1 N-RF1): AATCTTCTTGAACAGCCGCC	Integrated DNA Technologies	This paper

**Software and algorithms**		

GraphPad Prism v8	GraphPad software Inc.	https://www.graphpad.com/
ImageJ	National Institutes of Health	

### Resource availability

#### Lead contact

Further information and requests for resources and reagents should be directed to and will be fulfilled by the lead contact, Leonardo Salmena (leonardo.salmena@utoronto.ca).

#### Materials availability

The HEK293T and HeLa BRCA1-HiBiT cell lines created in this study are maintained at the University of Toronto and may be available upon request from the lead contact, Leonardo Salmena (leonardo.salmena@utoronto.ca).

#### Data and code availability

• Data and images generated as a part of this study are available upon request from the lead contact.
• This paper does not report original code.
• Any additional information required to reanalyze the data in this paper is available from the lead contact upon request.

### Experimental model and study participant details

#### Cell culture and treatments

HEK293T (transfection optimized), HeLa, U2OS, DU145, and breast cancer cell lines MDA-MB-231 and MCF7, were obtained from the American Type Culture Collection (ATCC, Manassas, VA, USA). HEK293T, HeLa, U2OS, MDA-MB-231, and MCF7 cells were cultured in Dulbecco’s Modified Eagle Medium (DMEM, Life Technologies, Burlington, ON, Canada) supplemented with 10% fetal bovine serum (FBS) and 100 units/mL penicillin and 100μg/mL streptomycin. DU145 cells were cultured in Roswell Park Memorial Institute 1640 medium (RPMI1640) Life Technologies, Burlington, ON, Canada) supplemented with 10% fetal bovine serum (FBS) and 100 units/mL penicillin and 100μg/mL streptomycin. All cells were maintained at 37°C in an atmosphere of 5% CO_2_. Cell treatments include differing concentrations of proteasome inhibitor MG132 (Millipore) from a stock concentration of 10mg/mL and translation inhibitor cycloheximide (Cell Signalling Technologies) from a stock concentration of 10mg/mL dissolved in dimethyl sulfoxide (DMSO).

### Method details

#### CRISPR Knock-in transfection

BRCA1-HiBiT fusion protein expressing cell lines were generated using CRISPR-mediated homology-directed repair to insert the 39bp HiBiT sequence in-frame, 1 codon upstream of the canonical stop codon of exon 24. Donor fragment is composed of homology arms of 40bp upstream and downstream of the insertion site were designed by Promega (5’ TGCCAGGAGCTGGACACCTACCTGATACCCCAGATCCCCCACAGCCACTACGTCTCCGTGAGCGGCTGGCGGCTGTTCAAGAAGATTAGCTGACTGCAGCCAGCCACAGGTACAGAGCCACAGGACCCCAAGAATGAGCT 3’) and purchased from Integrated DNA Technologies (IDT) as a single-stranded oligonucleotide donor (ssODN). A corresponding synthetic guide RNA (sgRNA) near the insertion site (5’ TACTGACTGCAGCCAGCCAC 3’) was also identified and purchased from IDT) as a pre-synthesized RNA fragment. The donor fragment as well as the sgRNA were delivered to cell lines using Thermofisher EnGen Cas9NLS protein (New England Biolabs) using Lipofectamine RNAiMax transfection reagent via reverse-transfection. Lyophilized sgRNA was reconstituted in RNAse-free water to a concentration of 24μM. EnGen Cas9 NLS protein was dissolved in glycerol at a stock concentration of 20μM. Donor arm ssODN template was prepared by diluting in RNAse-free, cell culture grade water to a concentration of 100μM. For preparation of RNP complexes, for transfection of biological triplicates in 96-well culture format: 5μL of 24μM sgRNA was combined with 5μLof 20μM Cas9 protein. 71.75μLof OptiMem Reduced Serum Media (Life Technologies) was added to ribonucleoprotein (RNP) complexes which were then incubated for 5 min at room temperature. 1 μL of 100μM HDR template was added to RNP complexes and 4.2μL Lipofectamine RNAiMax was added to each reaction with Optimem to a final volume of 175 μL. RNP-lipid complexes were incubated at RT for 20-30min. 50,000 HEK293T or HeLa cells per well were plated in 100μL. 50 μL of RNP and Lipofectamine mixture and 100 μL of cells were added to each well of a 96-well plate following which cells were incubated for 48 hours. Expression of HiBiT was determined by performing the Nano-Glo® HiBiT Lytic Assay on 20,000 transfected cells in triplicate.

#### Nano-Glo® HiBiT Lytic Assay

Cells were trypsinized, counted and plated in triplicate wells of CellStar 96 well white bottom, flat bottom microplates (Grenier Bio-One) and cells were allowed to adhere overnight in the cell culture incubator. Prior to the assay, cells and reagents were equilibrated to room temperature. HiBiT Lytic Reagent was prepared by diluting LgBiT protein 1:100 and the Nano-Glo® HiBiT Lytic Substrate 1:50 into room temperature Nano-Glo® HiBiT Lytic Buffer and mixed gently. Media was removed from cells. HiBiT Lytic Buffer was diluted 1:1 with 1XPBS and 50μL was dispensed onto wells in 96 well dishes and mixed by placing the plate on an orbital shaker at 350rpm for 20 minutes. Luminescence was measured using a Promega GloMax® Multi Detection Plate Reader or PerkinElmer EnVision 2105 Multimode Plate Reader using an integration time of 0.5 seconds.

#### Clonal selection

HiBiT positive pools were trypsinized and resuspended in FBS-containing complete media and counted using a Countess Automated Cell Counter (Invitrogen) using Trypan blue for dead cell discrimination. Cells were plated at low density as single cells (1000 cells in a 10cm plate) and allowed to grow to form colonies. Colonies were chosen and screened using the Nano-Glo® HiBiT Lytic Assay.

#### PCR screening and sequencing

For gnomic DNA extraction, cell pellets were resuspended in 500 μL DNA extraction buffer (1M Tris (pH 8.0), 20% sodium dodecyl sulfate (SDS), 0.5M EDTA, 5M NaCl_2_, dissolved in single-distilled water) and incubated for 16 hr shaking at 55°C. An equal volume of 100% EtOH was added, and DNA was centrifuged at 21,100 x g for 10 min. The supernatant was discarded, and the pellet washed in 500 μL 70% EtOH, followed by centrifugation for 10 min at 21,100 x g. The DNA pellet was air-dried for a minimum of 10 min and resuspended in nuclease-free water. PCR was performed using 1μL DNA of each clone, 1μL of forward primer, 1μL of reverse primer, 25μL of 2X Taq FroggaMix, and 22μL nuclease-free water. Primers used are detailed as follows: BRCA1 F1 Forward: 5’ GCAATTGGGCAGATGTGTGA 3’; BRCA1 R1: 5’ ACCCTTGCATAGCCAGAAGT 3’; BRCA1 N-F1: 5’ GGCGGCTGTTCAAGAAGATT 3’; BRCA1 N-R1: 5’ AATCTTCTTGAACAGCCGCC. 3’. PCR conditions are as follows: initial denaturation was performed at 95°C for 5 min followed by 35 cycles of 1 min denaturation at 95°C, 1 min annealing at 52°C for F1 and R1 primers, and 2 min extension at 72°C, followed by a final extension at 72°C for 10 min. PCR purification was performed using the QIAquick® PCR Purification Kit following manufacturer protocols. The expected fragment size of the BRCA1 F1 and R1 primers was 291 bp in parental size and 330 bp in targeted cells. Sanger sequencing was performed by ACGT Corporation (700 Bay Street, Toronto, ON), using the N-F1 and N-R1 primers.

#### siRNA transfection

siRNA knockdown was performed using siGENOME SMARTpool, a mixture of 4 Human BRCA1 siRNA (Horizon Discovery), and DharmaFECT Transfection Reagent (Horizon Discovery). siBRCA1 or siNTC (non-targeting control) was diluted to a working stock of 2μM. Dharmafect:siRNA complexes were added to 2 wells of a 12-well cell culture plate and allowed to incubate for 30 minutes at room temperature. Cells were trypsinized and 5 X 10^5^ cells were plated per well in 800μL in wells containing 200μL Dharmafect:siRNA. Cells were grown overnight. Cells were then moved to 6 well plates and media changed to complete media after 24 hours. Cells were allowed to incubate 48 hours before lysate collection and HiBiT Lytic Assay.

#### Taqman qRT-PCR

Total RNA was harvested using the RNeasy Mini Kit (Qiagen), using the manufacturer’s recommended method. cDNA was synthesized from 1μg total RNA using the SuperScript™ IV VILO™ with ezDNase Master Mix kit (Invitrogen™, ThermoFisher Scientific). DNase digestion was performed at 25°C for 10 min, reverse transcription was performed for 10 min at 50°C, and then the enzyme was deactivated at 85°C for 5 min. Following cDNA synthesis, qPCR analysis was performed using the TaqMan™ Fast Advanced Master Mix (Applied Biosystems™, ThermoFisher) on the QuantStudio 3 Real-Time PCR System (Applied Biosystems, ThemoFisher Scientific). TaqMan probes used are as follows: BRCA1 (Hs01556193_m1), Rad51 (Hs00947967), BRCA2 (Hs00609073), PALB2 (Hs00954121), Beta Actin (Hs01060665_g1), and GAPDH (Hs02786624_g1).

#### Immunoblotting and antibodies

Cells were lysed using 1 X Whole Cell Lysis with cOmplete™ EDTA-free protease inhibitor. Protein concentration of lysates was determined using the Bio-Rad Protein Assay and equal amounts of protein were prepared using WCL buffer, 5X Laemmli Buffer and resolved on a Bolt 4-12% Bis-Tris Plus Gel (Invitrogen), and then transferred to a nitrocellulose membrane overnight at 25V. Membranes were blocked using a solution of 5% skim milk in 1 X TBS-T and probed with antibodies. Membranes were blotted using a mouse anti-BRCA1 (1:250; Millipore Sigma, cat# OP92, MS110), rabbit anti-Rad51 (1:1000, CST, cat# 8875, D4B10), rabbit anti-BRCA2 (1:1000, CST, D9S6V), rabbit anti-PALB2 (1:500, CST, E9R2W) or a rabbit anti-GAPDH rabbit antibody (1:10000; CST, 2118). Secondary antibodies for anti-mouse-HRP (1:500; Kindle Biosciences, R1005) or anti-rabbit-HRP (1:10000; Kindle Biosciences, 1007) were added. Chemiluminescent reagent and the KwikQuant Imager (Kindle Biosciences) were used to visualize antibody binding.

#### Nano-Glo® HiBiT blotting system

Proteins were prepared, resolved, and transferred to a nitrocellulose membrane as above. Following transfer, membranes were incubated in 1X TBS-T for 15 min to solubilize the HiBiT tag. Nano-Glo® HiBiT Blotting Reagent was prepared by diluting the Nano-Glo® HiBiT Blotting Buffer tenfold in single-distilled water. LgBiT protein was diluted in 1X Nano-Glo® HiBiT Blotting Buffer 200-fold and mixed by inversion. The membrane was incubated with the Nano-Glo® HiBiT Blotting Reagent overnight at 4°C while shaking gently. Nano-Glo® Luciferase Assay Substrate was then diluted 500-fold in the LgBiT/buffer solution. The membrane was then incubated with the substrate for 5 min at room temperature while shaking gently and transferred to the iBright Western Blot Imaging System (Life Sciences) for imaging using the settings for luminescence and exposing for 30 min.

#### Alamar Blue viability assay

HEK293T, HeLa or MDA-MB-231 cells were plated for drug dosing in both white and clear parallel 96-well plates and allowed to adhere. After 24 hours, cells were treated with appropriate doses of drug to achieve desired final concentration as described above and incubated for the desired treatment time (12, 24, or 48 hours). After incubation, 10% by volume Alamar Blue Viability Reagent was added to all wells of clear plates. Cells were incubated for 4 hours protected from light at 37°C. Viability was assessed by reading the plate using the SpectraMax Spectrophotometer using an excitation wavelength of 530/560nm and emission wavelength of 590nm.

#### Crystal violet growth curve

Adherent cells were plated at a density of 2.5 X10^4^ cell/well in multiple 12-well plates. 24 hours after plating, cells were treated with appropriate doses of drug or DMSO. At various time points after treatment, cells were washed with PBS and fixed with 10% formalin Plates were stained with 0.1% Crystal Violet dissolved in 20% methanol then de-stained by washing twice with ultrapure water and allowed to dry. Quantification was performed by solubilizing crystal violet with 10% acetic acid and absorbance was determined at 595nM.

#### ɣH2A.X flow cytometry

MDA-MB-231 cells were plated for flow cytometry at a density of 5.0 X 10^5^ cells/well in individual wells of 6-well plates. 24 hours following plating, cells were treated with appropriate doses of DMSO or drug for 24 hours. In some cases, cells were exposed to 10Gy of γ-irradiation using the Gammacell 1000 Elite irradiator containing Cesium137 radionuclide and allowed to incubate for 6 hours. Following treatment, cells were trypsinized and fixed for 20min on ice with a 1:16 dilution of 37% formaldehyde containing 10-15% methanol. Cells were then washed in 1X PBS and permeabilized using a 1:10 dilution of 0.2μM filtered, 5% saponin, 100nM HEPES, pH 74, 1.4M NaCl, 25mM CaCl_2_ for 20min on ice with 3.5μL of either normal mouse IgG, FITC conjugate (in PBS with 5mg/mL BSA and 0.05% sodium azide) or 3.5μL of anti-phospho-Histone H2A.X (Ser139), FITC-conjugate (in PBS with 5mg/mL BSA and 0.05% sodium azide). Following incubation, cells were washed with a 1:10 dilution of 1% saponin in PBS to remove non-specific binding. Cells were then spun down and resuspended in PBS for flow cytometry analysis.

#### ɣH2A.X and Rad51 immunofluorescence

Prior to cell seeding, 15mmX15mm glass coverslips were coated with 0.01% poly-L-lysine (Sigma-Aldrich) in one well of a 12-well tissue culture dish and allowed to dry. MDA-MB-231 cells were plated for immunofluorescence (IF) at a density of 5.0 X 10^5^ cells/well in individual wells of 6-well plates. 24 hours following plating, cells were treated with appropriate drug doses for 24 hours. Following treatment, cells were fixed with 4% formaldehyde for 15 minutes, washed 3 times with 1X PBS, permeabilized with 0.3% Triton X-100, and washed 3 additional times. Cells were blocked using 3% bovine serum album (BSA) in 1X PBS for ɣH2A.X IF or 5% FBS, 0.2% Triton X-100, and 0.5% BSA in 1X PBS for Rad51 foci formation for 60min at room temperature.

For Rad51 IF, cells were treated with 8 Gy of IR. EdU staining was performed as per the product manual (Invitrogen, C10337). Following blocking, cells were incubated overnight at 4°C in primary antibody diluted in antibody binding buffer (1% BSA and 0.3% Triton X-100 in 1X PBS). Antibodies used were phospho-histone H2A.X (Ser139) rabbit primary antibody (Millipore Sigma, JBW301, y ) diluted 1:1000 and RAD51 rabbit primary (Abcam, ab176458) diluted 1:15000. Cells were washed three times with 1X PBS and incubated for 1hr at room temperature, in darkness with Goat anti-Rabbit IgG, Alexa Fluor™ 488 secondary antibody (Invitrogen, ThermoFisher Scientific) diluted 1:1000 for ɣH2A.X IF or anti-rabbit IgG conjugated with Alexa Fluor 594 (Invitrogen) for Rad51 in antibody binding buffer. Finally, cells were washed 3-4 times in 1X PBS, stained with DAPI and mounted on coverslips using DAKO fluorescent mounting media or Mowiol (Sigma-Aldrich). Immunofluorescent images for ɣH2A.X IF were taken via a ZEISS Axiolmager M2 Epifluorescence microscope as an AxioCam MRm CCD camera, using the AxioVision Software Version 4.8 at 20X magnification. Images for Rad51 foci formation were taken using a Leica (DM4000B) fluorescence microscope under 100× magnification. For the Rad51 image quantifications, >100 EdU positive cells were counted for all conditions from at least three independent experiments.

#### SynergyFinder 3.0 synergy analysis

Cells were plated in 6 X 10 or 7 X 7 dose-response matrices with either 7 or 10 doses of experimental BRCA1 downregulating drug and 6 or 7 doses of the PARP inhibitor (PARPi) Olaparib. Synergy screens were run as technical duplicates and biological triplicate experiments and normalized to dimethyl sulfoxide (DMSO)-treated controls. Synergy was determined by comparison between the observed drug combination response on cell viability as measured by alamarBlue against the expected response, assuming the two drugs do not interact with one another. Synergy was quantitated using the Zero-Interaction Potency (ZIP) model, which integrates the Loewe additivity model and the Bliss independence model^96^ using the SynergyFinder web application (version 3.0).^97^ The ZIP method of quantification measures the difference in dose-response curves of individual drugs and the combination to evaluate changes in potency.^96^

#### HEK293T and HeLa BRCA1-HiBiT luminescence screens

High-throughput drug screens were performed at the High-Throughput Screening Facility at the Network Biology Collaborative Centre in Mount Sinai Hospital in Toronto, Canada. Prior to cell seeding, clear bottom 96- or 384-well white opaque plates (Grenier Bio-One CellStar), were coated with poly-L-lysine (Sigma-Aldrich). In the pilot epigenetic screen only, BRCA1-HiBiT reporter cells were then seeded in prepared white plates and parallel black 96-well plates (Sarstedt). HEK293T cells were then seeded at a density of 70,000 cells/mL resulting in 3500 cells in 50μL of media in a 96-well plate and 700 cells in 10μL of media in a 384-well plate. HeLa cells were seeded at a density of 40,000 cells/mL resulting in 2000 cells in 50μL of media in a 96-well plate. Cells were then incubated for 24hr in a 37°C cell culture incubator.

Following this, screening drugs were dispensed using the Biomek FX liquid handler (Beckman-Coulter). Drugs were dissolved in 50μL media at the appropriate concentrations (<0.5% DMSO). 0.25μM MG132 was used as a positive control in HEK293T screens, and gemcitabine was used as a positive control in HeLa screens. DMSO alone was used as a negative control. In the luminescence plates, control wells were also treated with HiBiT lytic buffer with or without the LgBiT recombinant protein as a control for background luminescence. Cells were then incubated for 24hr in a 37°C cell culture incubator.

The following day, cell viability was assessed in the black plate by alamarBlue Cell Viability Reagent (Invitrogen) as previously described. BRCA1-HiBiT expression was assessed by Nano-Glo® HiBiT Lytic Assay (Promega) as previously described. alamarBlue fluorescence and HiBiT luminescence were read using the EnVision plate reader (Perkin Elmer). Z-factor was calculated as a means of assessing variability and the screen was considered highly screenable if the Z-factor was upwards of 0.7. Hits were determined by B-score calculation.

#### Hit determination by B-Score calculation

Luminescence and cell viability screen hits were determined by B-score calculation. B-score (or B-statistic) is a statistical measure used in high-throughput screening (HTS) to assess the significance of the observed effects. It helps identify "hits'' or compounds that have a significant impact compared to control conditions by measuring the relative potency of the experimental data in comparison to the raw sample data.^34^^,^^35^ B-score is calculated similarly to Z-score as it is a ratio of the adjusted raw data divided by the variability across the plate.^34^^,^^35^ The raw data numerator is adjusted for row and column effects as well as plate-to-plate effects, and the variability value of the denominator is calculated from the residual variability in the plate after row and column effects are adjusted and smoothed.^34^ A high positive B-score indicates that the sample response is significantly higher than the control, suggesting it may be a potential hit or have a meaningful effect. Conversely, a low or negative B-score suggests that the sample response is not significantly different from the control. Compounds were considered hits if the corresponding B-score value for that well was 3 standard deviations away from the negative control value.

#### HEK293T AlamarBlue Cell viability sub-screen

Following the completion of the Medical Collection screen, a cell viability sub-screen was performed using the top 163 downregulating compounds. HEK293T BRCA1-HiBiT cells were seeded as previously described in clear bottom, black 384-well plates. Cells were then incubated for 24hr in a 37°C cell culture incubator. Drugs were then dispensed using the Biomek FX liquid handler (Beckman-Coulter). Drugs were dissolved in 50μL media at the appropriate concentrations as used in the primary screen (<0.5% DMSO). DMSO was used as a negative control. Cells were then incubated for 24hr in a 37°C cell culture incubator. The following day, cell viability was assessed by alamarBlue Cell Viability Reagent (Invitrogen) as previously described and read using the EnVision plate reader (Perkin Elmer).

#### MDA-MB-231 olaparib combination sub-screen

Prior to screen initiation, the 35 experimental drugs were serially diluted 4 times from high to low concentration at 1:4 dilution. MDA-MB-231 breast cancer cells were seeded at a density of 35,000 cells/mL resulting in 1,750 cells in 50μL of media in 4 clear bottom black 384-well plates. Cells were then incubated for 24hr in a 37°C cell culture incubator. The following day, DMSO or olaparib was dispensed using the Biomek FX liquid handler (Beckman-Coulter). 2 plates each were dosed with DMSO or olaparib at 5μM, a concentration that was previously established to reduce the growth of MDA-MB-231 cells by approximately 10% at 72 hrs post-treatment. Cells were then incubated for 1 hour. Cells were then dosed with one of the diluted 35 exploratory drugs. (+)-JQ1 and panobinostat were used as positive controls for combination with olaparib, and DMSO was used as a negative control. Cells were then incubated in a 37°C cell culture incubator for 72 hrs. Following this, cell viability was assessed by alamarBlue Cell Viability Reagent (Invitrogen) as previously described. Fluorescence was read using the EnVision plate reader (Perkin Elmer). Dose-response curves were then generated for each drug in the presence or absence of olaparib, and the area-under-the-curve was calculated to quantify the combination of the two drugs.

#### MDA-MB-231 olaparib synergy screen

Prior to screen initiation, dilution plates of olaparib and the seven experimental drugs (N-acetyl-N-acetoxy-4-chlorobenzenesulfonamide, IMD 0354, SNS-032, A-443654, ryuvidine, CHIR-124, and strophantine octahydrate) were prepared. 6 concentrations of each drug and olaparib were serially diluted 1:2, resulting in a 6X6 matrix of differing drug and olaparib combinations, with olaparib doses running horizontally and experimental drug doses running vertically. Each drug combination was plated in duplicate wells. MDA-MB-231 breast cancer cells were then seeded at a density of 35,000 cells/mL resulting in 1750 cells in 50μL of media in 4 clear bottom black 384-well plates. Cells were then incubated for 24hr in a 37°C cell culture incubator. The following day, the 6 olaparib doses were dispensed using the Biomek FX liquid handler (Beckman-Coulter). Cells were then incubated for 1 hour. Following this, the 6 experimental drug doses were dispensed. Panobinostat was used as a positive control for synergy with olaparib and DSMO was used as a negative control. Cells were then incubated in a 37°C cell culture incubator for 72 hrs. Following this, cell viability was assessed by alamarBlue Cell Viability Reagent (Invitrogen) as previously described. Fluorescence was read using the EnVision plate reader (Perkin Elmer). Synergy was then quantitated using the SynergyFinder 3.0 webtool as previously described.

### Quantification and statistical analysis

A one-way ANOVA was used for data groups that had the same single group factor in common, including but not limited to qRT-PCR, luminescence analysis, and alamarBlue cell viability. All data points were included. A Dunnett post-hoc test was performed when compared only to control and a Tukey post-hoc test was performed when comparing the different groups. Two-way ANOVAs were conducted for 2-factor analyses of groups, including growth curve analysis and area-under-the-curve quantitation. All data points were included. A Dunnett post-hoc was used when compared only to control, a Tukey post-hoc was performed when comparing between the different groups, and a Sidak post-hoc was performed on AUC data. All analyses were performed in GraphPad Prism v8. ∗ p<0.05, ∗∗ p<0.01, ∗∗∗ p<0.001, ∗∗∗∗ p<0.0001, ns, non-significant as shown in figures and figure legends.

## Supplemental information

Document S1. Figures S1–S9

Table S1. List of 163 HEK293T BRCA1-HiBiT Medical Collection, related to Figure 5 and Table 4
