# Supplementary material for: A high-throughput approach to identify BRCA1-downregulating compounds to enhance PARP inhibitor sensitivity
Source: iScience. 2024 Jun 4;27(7):110180. doi: 10.1016/j.isci.2024.110180 (PMC11238136; doi:10.1016/j.isci.2024.110180)
Supplement: Document S1. Figures S1–S9 [file mmc1.pdf]

iScience, Volume *27*

Supplemental information

A high-throughput approach to identify BRCA1-downregulating compounds to enhance PARP inhibitor sensitivity

Erin Sellars, Margarita Savguira, Jie Wu, Sabrina Cancelliere, Mark Jen, Rehna Krishnan, Anne Hakem, Dalia Barsyte-Lovejoy, Razqallah Hakem, Steven A. Narod, Joanne Kotsopoulos, and Leonardo Salmena

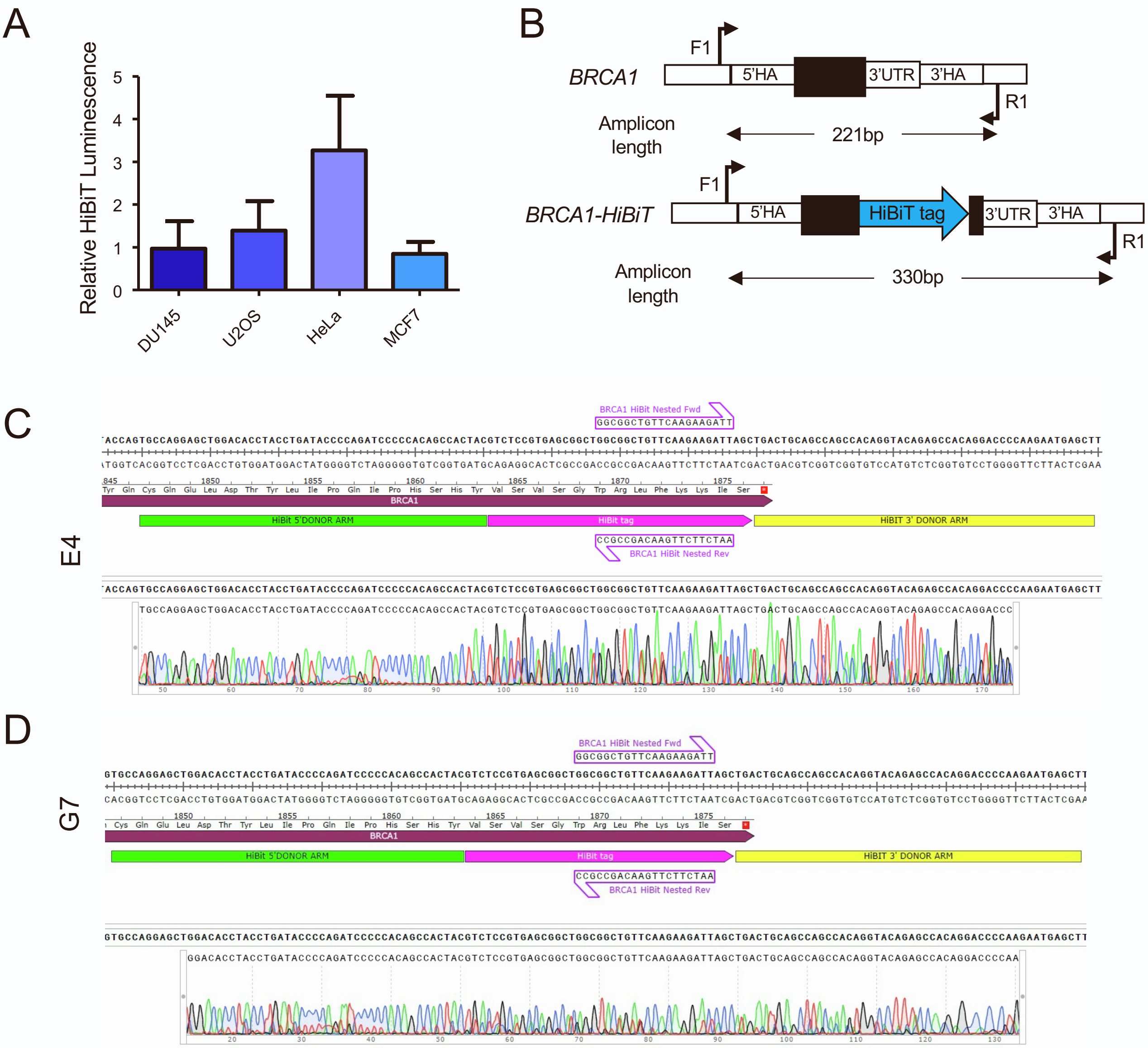
A
Relative HiBiT Luminescence
0
1
2
3
4
5
DU145
U2OS
HeLa
MCF7
B
BRCA1
F1
5'HA
3'UTR
3'HA
R1
Amplicon length
221bp
BRCA1-HiBiT
F1
5'HA
HiBiT tag
3'UTR
3'HA
R1
Amplicon length
330bp
C
E4
BRCA1 HiBit Nested Fwd
GGCGGCTGTTCAAGAAGATT
BRCA1
HiBit 5'DONOR ARM
HiBit tag
HiBIT 3' DONOR ARM
CCGCCGACAAGTTCTTCTAA
BRCA1 HiBit Nested Rev
D
G7
BRCA1 HiBit Nested Fwd
GGCGGCTGTTCAAGAAGATT
BRCA1
HiBit 5'DONOR ARM
HiBit tag
HiBIT 3' DONOR ARM
CCGCCGACAAGTTCTTCTAA
BRCA1 HiBit Nested Rev

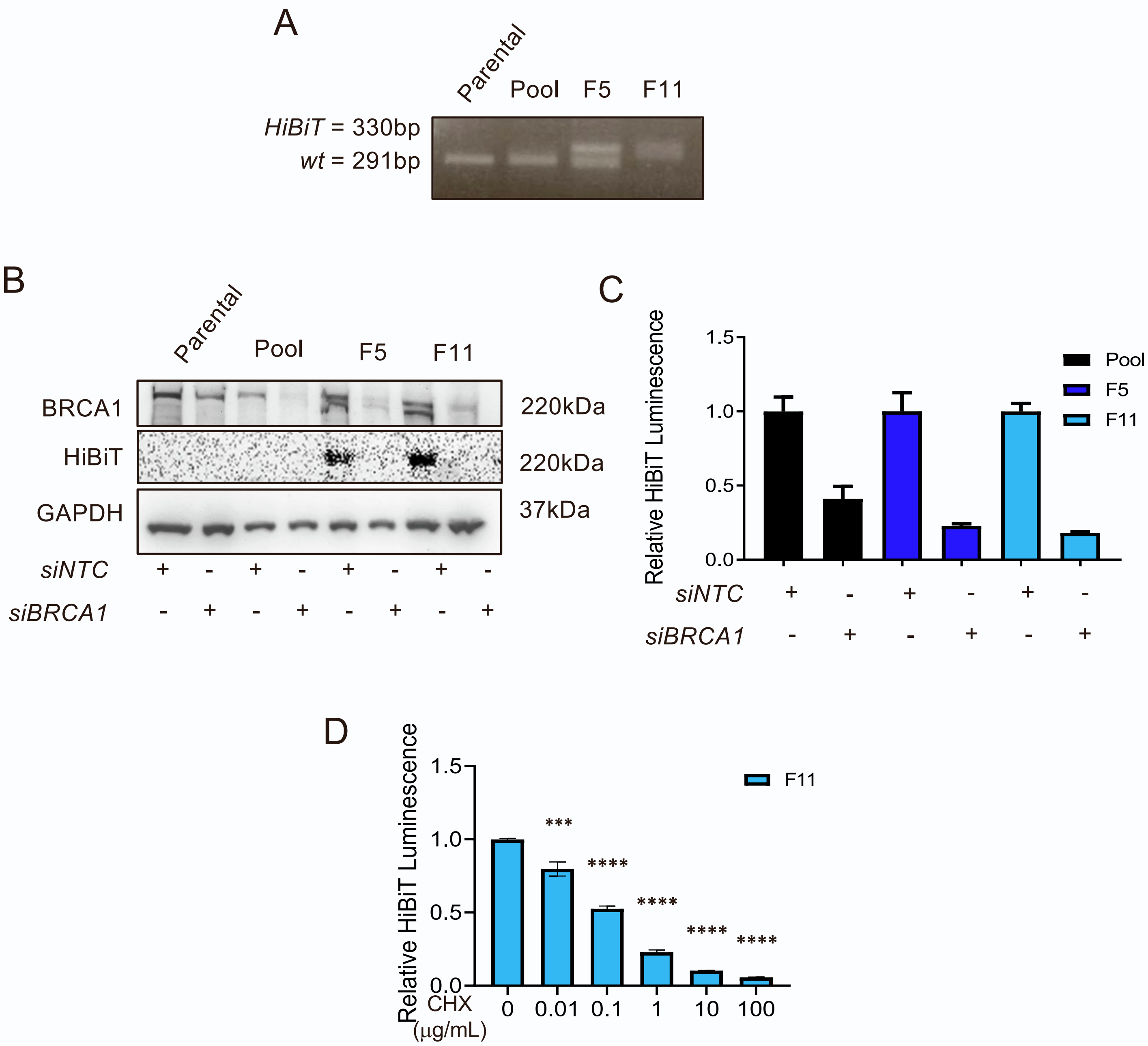
A
Parental
Pool
F5
F11
HiBiT = 330bp
wt = 291bp
B
Parental
Pool
F5
F11
BRCA1
HiBiT
GAPDH
220kDa
220kDa
37kDa
siNTC
siBRCA1
C
Relative HiBiT Luminescence
1.5
1.0
0.5
0.0
Pool
F5
F11
siNTC
siBRCA1
D
Relative HiBiT Luminescence
1.5
1.0
0.5
0.0
F11
***
****
****
****
****
CHX (μg/mL)
0
0.01
0.1
1
10
100

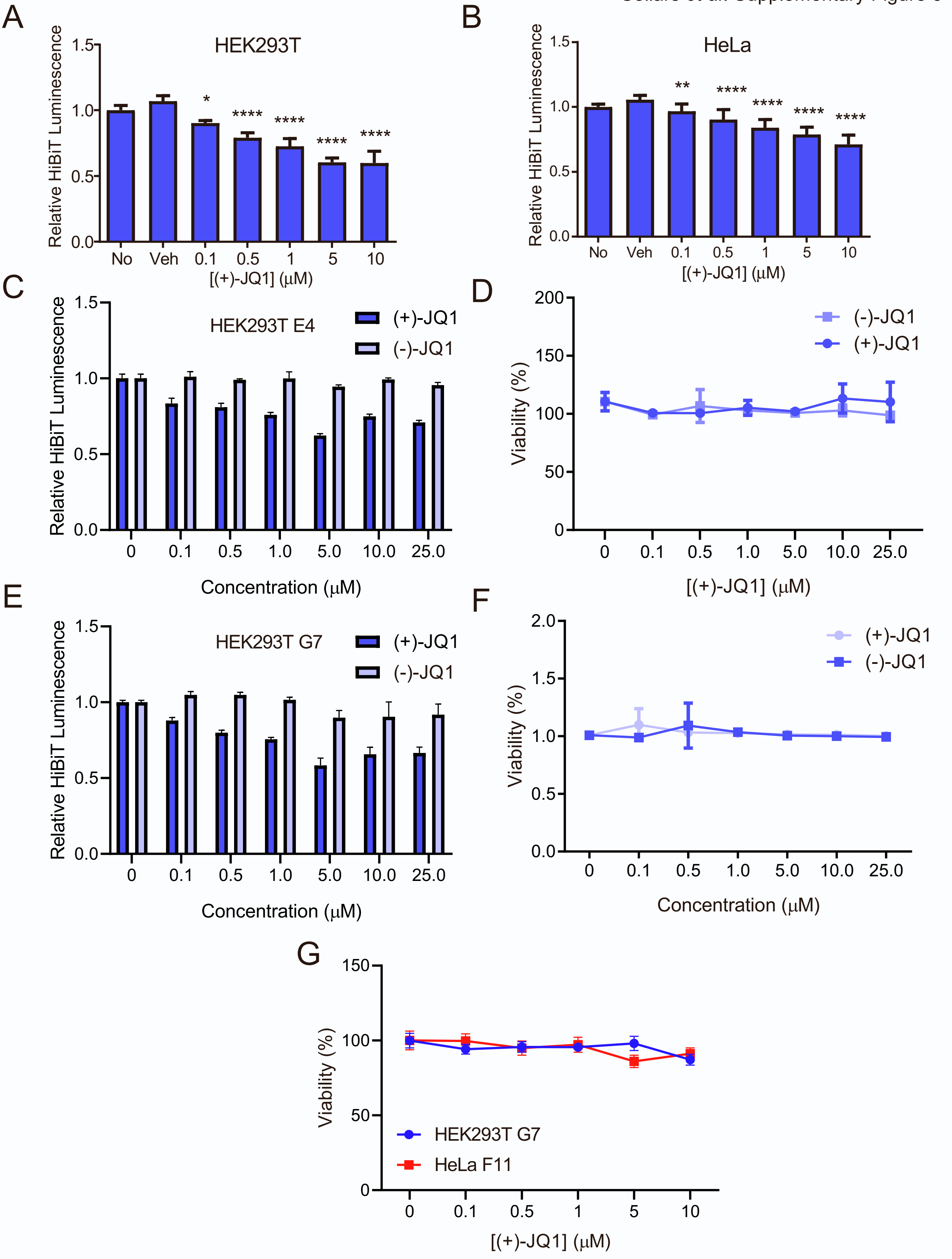
A
HEK293T
Relative HiBiT Luminescence
1.5
1.0
0.5
0.0
*
****
****
****
****
No
Veh
0.1
0.5
1
5
10
[(+)-JQ1] (µM)
B
HeLa
Relative HiBiT Luminescence
**
****
****
****
****
No
Veh
0.1
0.5
1
5
10
[(+)-JQ1] (µM)
C
HEK293T E4
(+)-JQ1
(-)-JQ1
Relative HiBiT Luminescence
0
0.1
0.5
1.0
5.0
10.0
25.0
Concentration (µM)
D
(-)-JQ1
(+)-JQ1
Viability (%)
200
150
100
50
0
[(+)-JQ1] (µM)
E
HEK293T G7
(+)-JQ1
(-)-JQ1
Relative HiBiT Luminescence
Concentration (µM)
F
(+)-JQ1
(-)-JQ1
Viability (%)
2.0
1.5
1.0
0.5
0.0
Concentration (µM)
G
Viability (%)
150
100
50
0
HEK293T G7
HeLa F11
0
0.1
0.5
1
5
10
[(+)-JQ1] (µM)

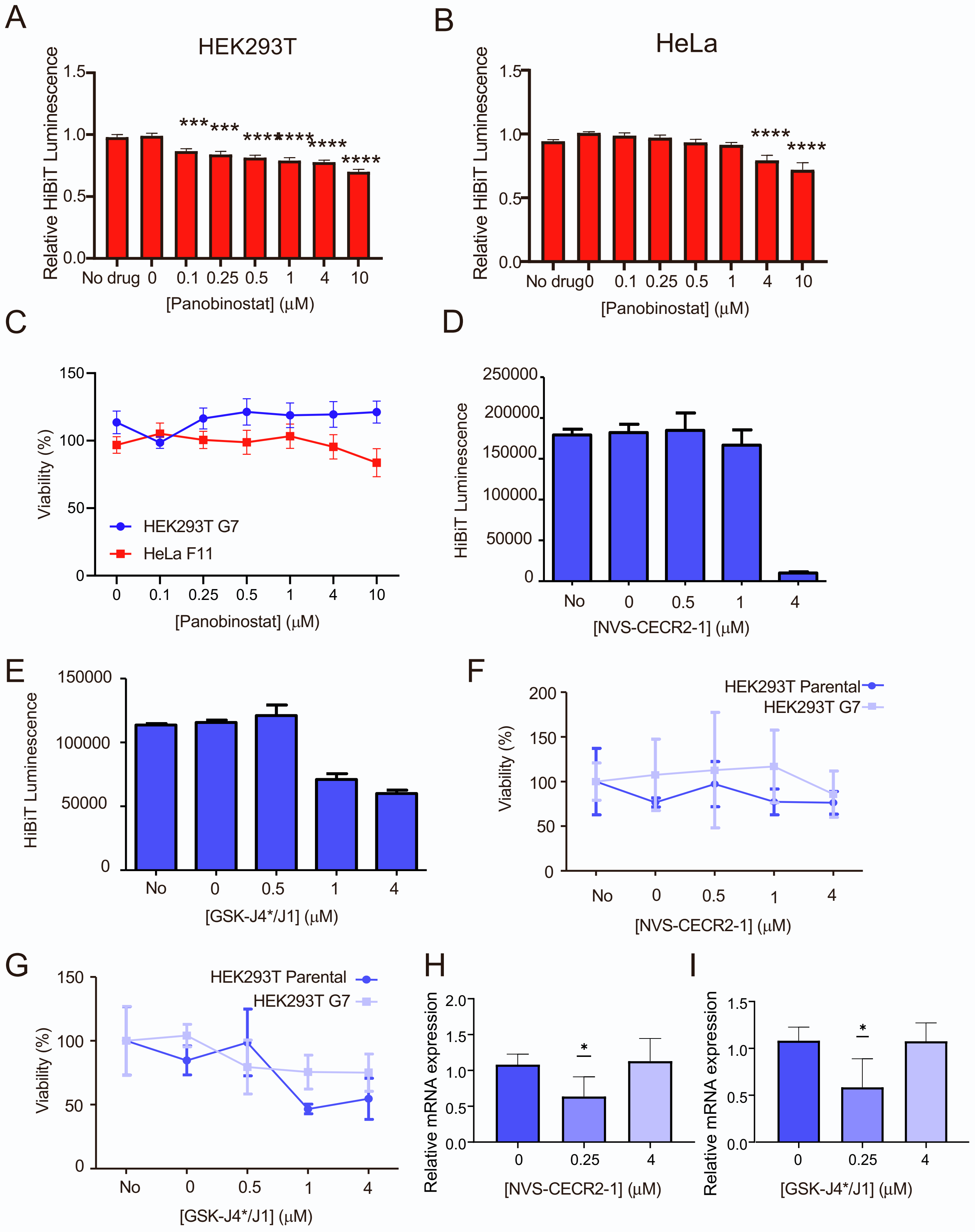
A
HEK293T
Relative HiBiT Luminescence
1.5
1.0
0.5
0.0
***
***
****
****
****
****
No drug 0 0.1 0.25 0.5 1 4 10
[Panobinostat] (μM)
B
HeLa
Relative HiBiT Luminescence
1.5
1.0
0.5
0.0
****
****
No drug 0 0.1 0.25 0.5 1 4 10
[Panobinostat] (μM)
C
Viability (%)
150
100
50
0
HEK293T G7
HeLa F11
0 0.1 0.25 0.5 1 4 10
[Panobinostat] (μM)
D
HiBiT Luminescence
250000
200000
150000
100000
50000
0
No 0 0.5 1 4
[NVS-CECR2-1] (μM)
E
HiBiT Luminescence
150000
100000
50000
0
No 0 0.5 1 4
[GSK-J4*/J1] (μM)
F
HEK293T Parental
HEK293T G7
Viability (%)
200
150
100
50
0
No 0 0.5 1 4
[NVS-CECR2-1] (μM)
G
HEK293T Parental
HEK293T G7
Viability (%)
150
100
50
0
No 0 0.5 1 4
[GSK-J4*/J1] (μM)
H
Relative mRNA expression
2.0
1.5
1.0
0.5
0.0
*
0 0.25 4
[NVS-CECR2-1] (μM)
I
Relative mRNA expression
1.5
1.0
0.5
0.0
*
0 0.25 4
[GSK-J4*/J1] (μM)

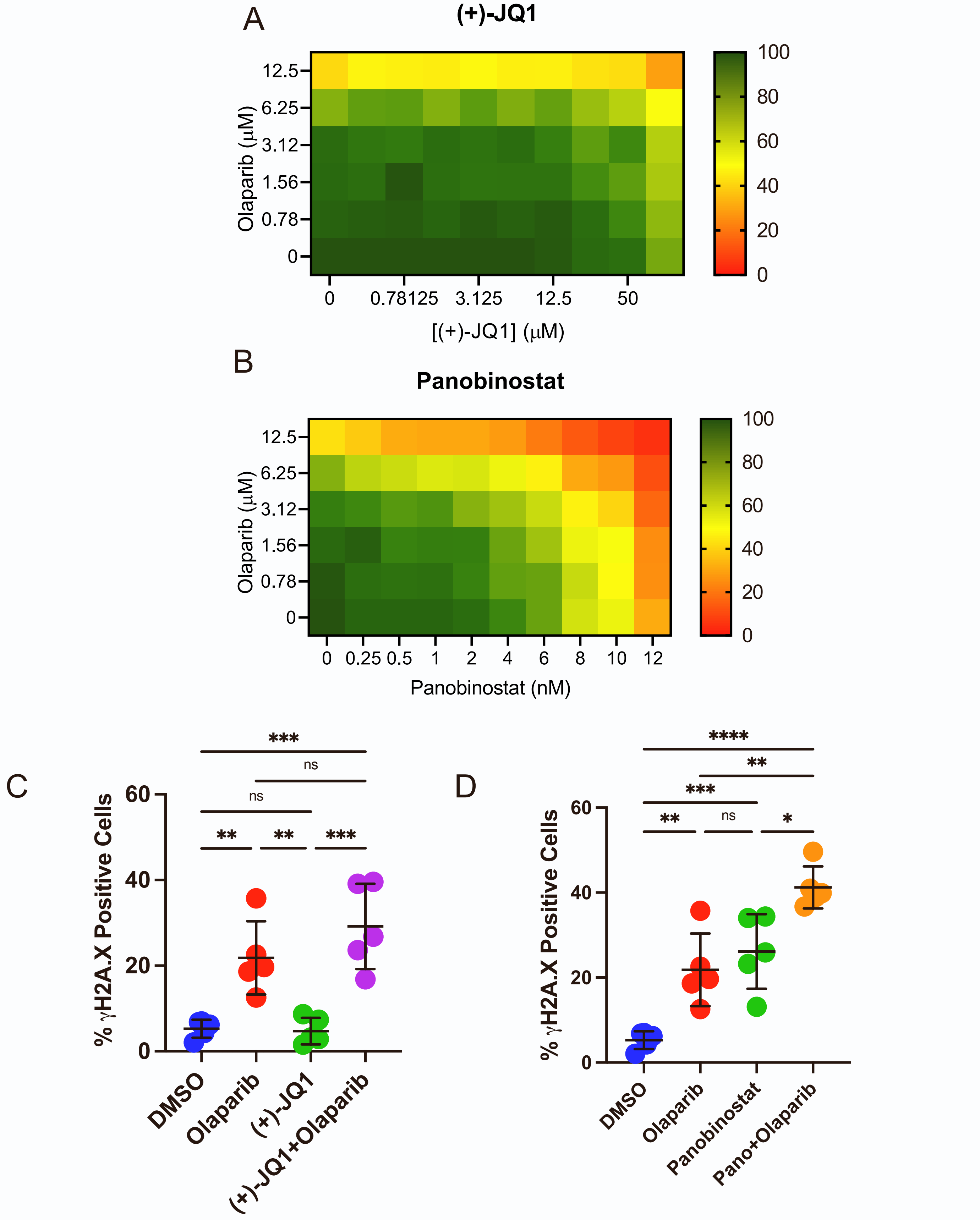
A
(+)-JQ1
12.5
6.25
3.12
1.56
0.78
0
Olaparib (μM)
0
0.78125
3.125
12.5
50
[(+)-JQ1] (μM)
100
80
60
40
20
0
B
Panobinostat
12.5
6.25
3.12
1.56
0.78
0
Olaparib (μM)
0
0.25
0.5
1
2
4
6
8
10
12
Panobinostat (nM)
100
80
60
40
20
0
C
% γH2A.X Positive Cells
60
40
20
0
***
ns
ns
**
**
***
DMSO
Olaparib
(+)-JQ1
(+)-JQ1+Olaparib
D
% γH2A.X Positive Cells
60
40
20
0
****
**
***
**
ns
*
DMSO
Olaparib
Panobinostat
Pano+Olaparib

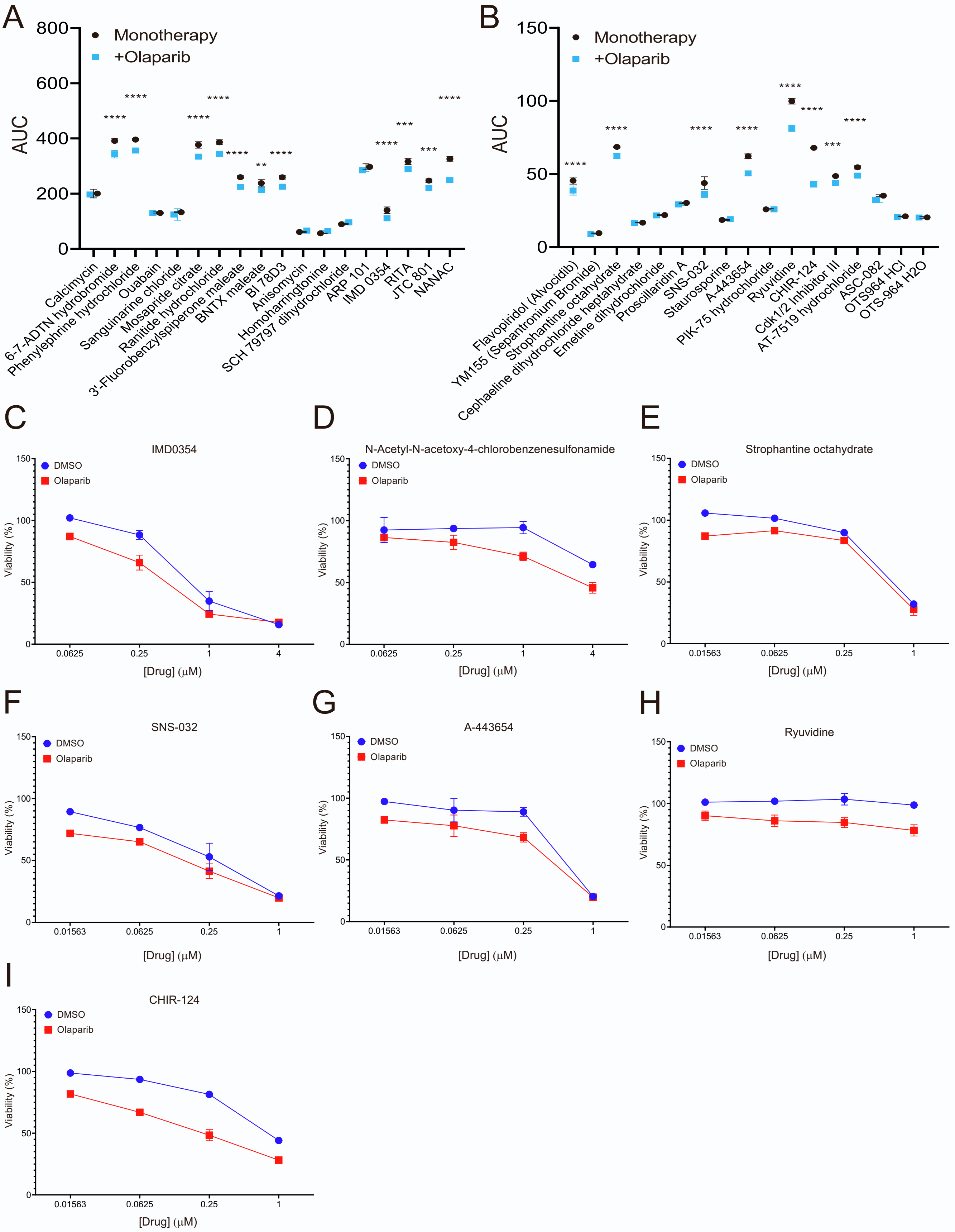
A
B
C
D
E
F
G
H
I
Monotherapy
+Olaparib
AUC
Calcimycin
6-7-ADTN hydrobromide
Phenylephrine hydrochloride
Ouabain
Sanguinarine chloride
Mosapride citrate
Ranitide hydrochloride
3'-Fluorobenzylspiperone maleate
BNTX maleate
BI 78D3
Anisomycin
Homoharringtonine
SCH 79797 dihydrochloride
ARP 101
IMD 0354
RITA
JTC 801
NANAC
Flavopiridol (Alvocidib)
YM155 (Sepantronium Bromide)
Strophantine octahydrate
Cephaeline dihydrochloride heptahydrate
Emetine dihydrochloride
Proscillaridin A
SNS-032
Staurosporine
A-443654
PIK-75 hydrochloride
Ryuvidine
CHIR-124
Cdk1/2 Inhibitor III
AT-7519 hydrochloride
ASC-082
OTS964 HCl
OTS-964 H2O
IMD0354
N-Acetyl-N-acetoxy-4-chlorobenzenesulfonamide
Strophantine octahydrate
SNS-032
A-443654
Ryuvidine
CHIR-124
DMSO
Olaparib
Viability (%)
[Drug] (μM)

Sellars *et al*. Supplementary Figure 7

A

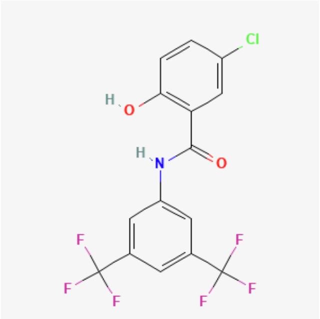

B

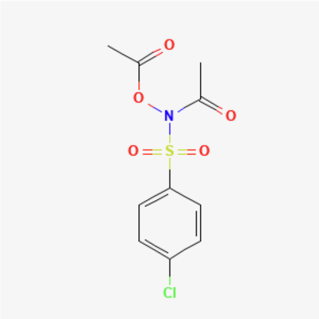

C

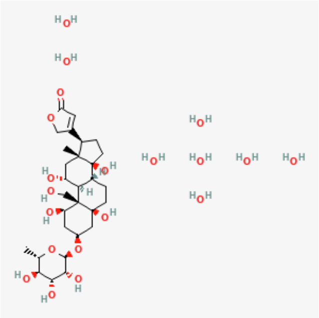

D

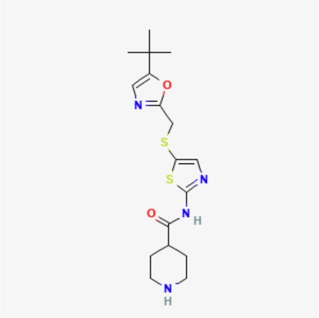

E

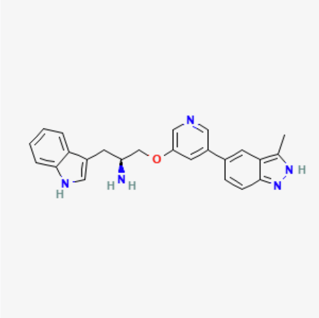

F

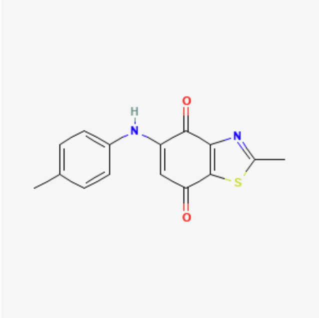

G

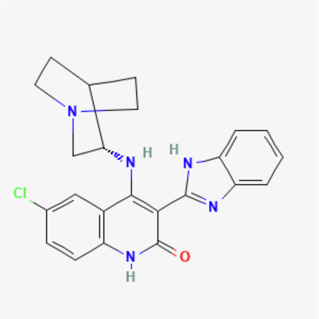

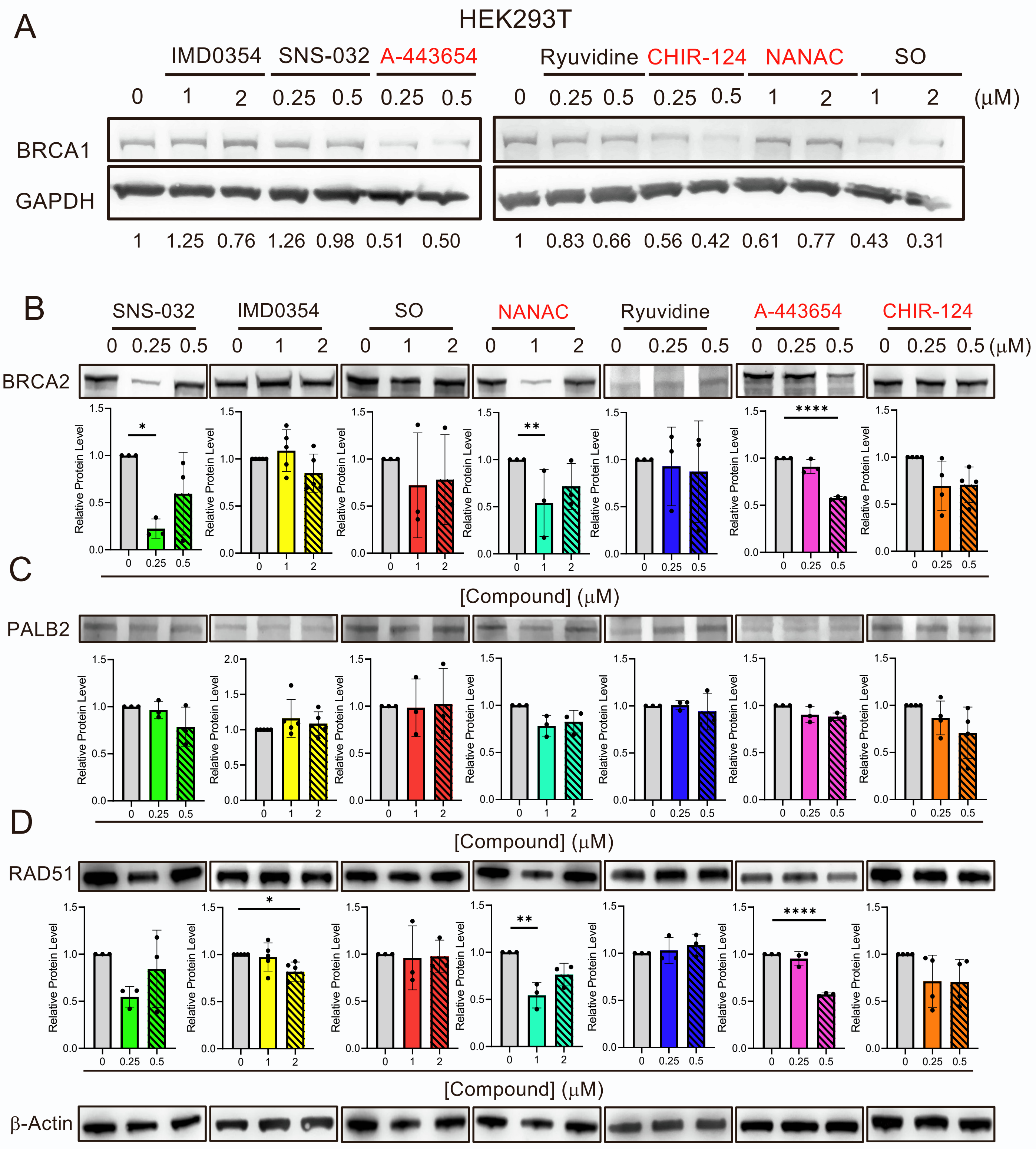
A
HEK293T
IMD0354
SNS-032
A-443654
Ryuvidine
CHIR-124
NANAC
SO
(μM)
BRCA1
GAPDH
1 1.25 0.76 1.26 0.98 0.51 0.50
1 0.83 0.66 0.56 0.42 0.61 0.77 0.43 0.31
B
BRCA2
Relative Protein Level
[Compound] (μM)
C
PALB2
D
RAD51
β-Actin

Sellars *et al*. Supplementary Figure 9

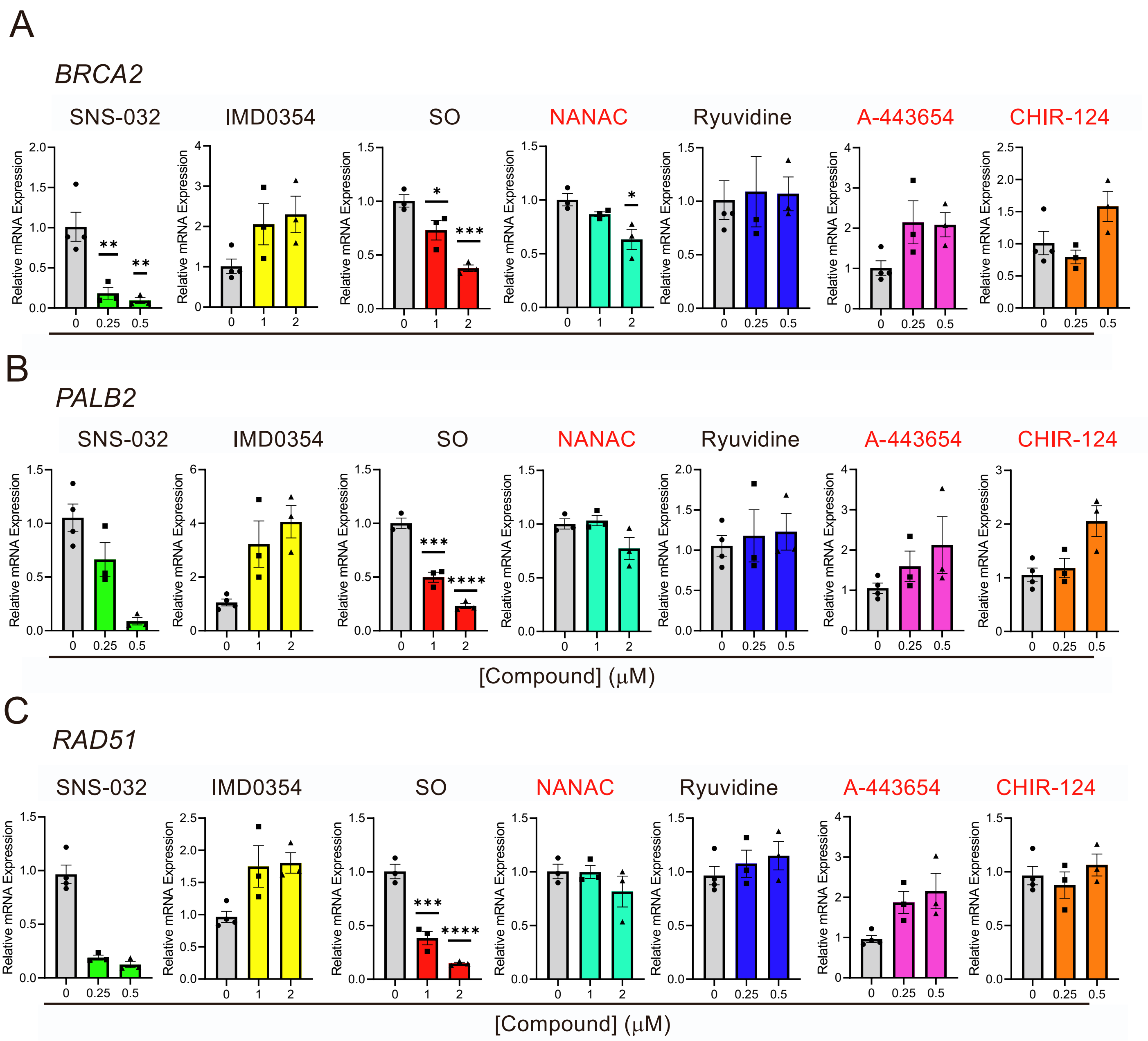

SUPPLEMENTARY FIGURE TITLES AND LEGENDS

Supplementary Figure 1. Generating HEK293T and HeLa BRCA1-HiBiT reporter cells. Related to Figure 1. a) DU145, U2OS, HeLa, and MCF7 cells were transfected with BRCA1-HiBiT CRISPR/Cas9 and luminescence measured after 48 hrs. **b)** BRCA1-HiBiT PCR screen primer schematic and expected fragment sizes. **c,d)** Sequence map and sanger sequencing of the targeted BRCA1 locus in HEK293T BRCA1-HiBiT clones E4 and G7.

Supplementary Figure 2. Validation of HeLa BRCA1-HiBiT reporter cells. Related to Figures 1 and 2. a) PCR screening of HeLa BRCA1-HiBiT pool, F5, and F11 clones. **b)** Western blot and HiBiT blot of parental HeLa, HiBiT targeted HeLa pool, and HeLa-HiBiT clones F5 and F11 transfected with BRCA1 siRNA or NTC siRNA. **c)** Histograms demonstrating HiBiT luminescence in HiBiT targeted HeLa pool, and HeLa-HiBiT clones F5 and F11 transfected with BRCA1 siRNA or NTC siRNA. HeLa cells. However, **d)** Histograms demonstrating HiBiT luminescence in HeLa-HiBiT F11 E4 clone after treatment with depicted doses of cycloheximide for 24hr. Relative HiBiT Luminescence refers to light units normalized to DMSO treated control within the F11 cell line. All replicate experiments were performed at least three times and representative images shown.

Supplementary Figure 3. Validation of (+)-JQ1 in HEK293T and HeLa BRCA1-HiBiT cells. Related to Figure 4. (+)-JQ1 significantly reduced BRCA1 HiBiT luminescence in **a)** HEK293T BRCA1-HiBiT and **b)** HeLa F11 BRCA1-HiBiT reporter cells in a dose-dependent manner. **c, d**) (+)-JQ1 but not (-)-JQ1 reduced BRCA1-HiBiT luminescence in HEK293T E4 and **e, f)** G7 cells with no change in cell viability. **g)** Increasing doses of (+)-JQ1 did not significantly affect cell viability in HEK293T G7 or HeLa F11 BRCA1-HiBiT reporter cells. All replicate experiments were performed at least three times and representative images shown.

Supplementary Figure 4. Validation of panobinostat in HEK293T and HeLa BRCA1-HiBiT cells. Related to Figure 4. Panobinostat significantly reduced BRCA1 HiBiT luminescence in **a)** HEK293T G7 and **b)** HeLa F11 reporter cells in a dose-dependent manner. **c)** Increasing doses of panobinostat did not significantly affect cell viability in HEK293T G7 or HeLa F11 BRCA1-HiBiT reporter cells. **d)** NVS-CECR2-1 reduced BRCA1-HiBiT luminescence in HEK293T G7 cells, at the 4 μM dose only. **f)** NVS-CECR2-1 did not affect viability in HEK293T cells at 24 hrs. **h)** NVS-CECR2-1 reduced BRCA1 mRNA expression in MDA-MB-231 cells at the 0.25 μM dose only, but not at 4 μM. **e)** GSK-J4*/J1 reduced BRCA1-HiBiT luminescence in HEK293T G7 cells, at the 1 μM, and 4 μM doses. **g)** GSK-J4*/J1 reduced cell viability by approximately 50% in HEK293T parental and G7 cells at the 1 μM, and 4 μM doses. **i)** GSK-J4*/J1 reduced BRCA1 mRNA expression in MDA-MB-231 cells at the 0.25 μM, but not at 4 μM. All replicate experiments were performed at least three times.

Supplementary Figure 5. Validation of (+)-JQ1 and panobinostat in MDA-MB-231 cells. Related to Figure 4. a,b) MDA-MB-231 cells were treated in a 10 x 6 matrix of increasing **a)** (+)-JQ1 + olaparib dose combinations, **b)** or increasing panobinostat + olaparib dose combinations, and cell viability was measured and plotted as a viability heatmap with Synergy Finder. In MDA-MB-231 cells were treated with 5 μM **c)** (+)-JQ1 or **d)** 4 μM panobinostat with or without 10 μM olaparib for 24 hrs, gH2A.X flow cytometry was performed. All replicate experiments were performed at least three times.

Supplementary Figure 6. AUC analysis of compounds from the Medical Collection. Related to Figure 5. AUC analysis of compounds from the **a)** LOPAC, NIH1, NIH2, and TOCRIS and **b)** PKI, Selleck, and Prestwick sub-libraries. Individual drugs were tested at 4 doses and a single dose of olaparib was used (5 μM]. Dose-response curves for cell viability were generated for each compound as a monotherapy and in combination with olaparib. Area under the curve (AUC) was

calculated for each compound and drugs were selected that significantly reduced cell viability in conjunction with olaparib. Dose-response curves for **c)** IMD-0354, **d)** NANAC, **e)** strophantine octahydrate (SO), **f)** SNS-032, **g)** A-443543, **h)** ryuvidine, and **i)** CHIR-124. MDA-MB-231 cells were assessed for cell viability following treatment with individual drugs as monotherapy and in conjunction with olaparib.

Supplementary Figure 7. Candidate compounds. Related to Figure 6. Chemical structures for **a)** IMD-0354**, b)** NANAC, **c)** SO, **d)** SNS-032, **f)** A-443543, **g)** ryuvidine, and **g)** CHIR-124 are depicted.

Supplementary Figure 8. Validation of candidate compounds by Western blot. Related to Figure 7. a) Western blot of HEK293T cells treated with candidate compounds. Western blots for **b**) BRCA2, **c**) PALB2 and **c**) RAD51 proteins accompanied by quantitation of three independent experiments in MDA-MB-231 cells treated with candidate compounds. Beta Actin was used as loading control. All replicate experiments were performed at least three times and representative images shown.

Supplementary Figure 9. Validation of candidate compounds by qRT-PCR. Related to Figure 7. qRT-PCR analysis showed that SNS-032 and SO significantly downregulated expression of **a)** *BRCA2*, **b)** *PALB2* and **d)** *Rad51* transcripts. NANAC reduced expression of *BRCA2* transcript levels only at the higher dose. A-443654 and CHIR-124 did not significantly reduce the expression of any HR-associated transcripts tested. All replicate experiments were performed at least three times.
